# Pesticide Aptasensors—State of the Art and Perspectives

**DOI:** 10.3390/s20236809

**Published:** 2020-11-28

**Authors:** Kamonrat Phopin, Tanawut Tantimongcolwat

**Affiliations:** 1 Center for Research and Innovation, Faculty of Medical Technology, Mahidol University, Nakorn Pathom 73170, Thailand; kamonrat.php@mahidol.ac.th; 2 Department of Clinical Microbiology and Applied Technology, Faculty of Medical Technology, Mahidol University, Bangkok 10700, Thailand

**Keywords:** aptamers, SELEX, pesticides, biosensors, ASSURED

## Abstract

Contamination by pesticides in the food chain and the environment is a worldwide problem that needs to be actively monitored to ensure safety. Unfortunately, standard pesticide analysis based on mass spectrometry takes a lot of time, money and effort. Thus, simple, reliable, cost-effective and field applicable methods for pesticide detection have been actively developed. One of the most promising technologies is an aptamer-based biosensor or so-called aptasensor. It utilizes aptamers, short single-stranded DNAs or RNAs, as pesticide recognition elements to integrate with various innovative biosensing technologies for specific and sensitive detection of pesticide residues. Several platforms for aptasensors have been dynamically established, such as colorimetry, fluorometry, electrochemistry, electrochemiluminescence (ECL) and so forth. Each platform has both advantages and disadvantages depending on the purpose of use and readiness of technology. For example, colorimetric-based aptasensors are more affordable than others because of the simplicity of fabrication and resource requirements. Electrochemical-based aptasensors have mainly shown better sensitivity than others with exceedingly low detection limits. This paper critically reviews the progression of pesticide aptasensors throughout the development process, including the selection, characterization and modification of aptamers, the conceptual frameworks of integrating aptamers and biosensors, the ASSURED (affordable, sensitive, specific, user-friendly, rapid and robust, equipment-free and deliverable to end users) criteria of different platforms and the future outlook.

## 1. Introduction

The world population is currently suffering from toxic pollutant exposure and one of the major issues is contamination by toxic agrochemicals in food chains and living environments, such as pesticides, antibiotics and fertilizers, among which pesticides are responsible for the highest prevalence of adulteration and toxicity. Contaminated foods and drinks are the main sources of pesticide dissemination that result in serious health issues for people. The maximum allowable levels of pesticide residues in agricultural products have been legally issued in terms of maximum residue limits (MRLs) or tolerance limits according to the consolidation among types of pesticides and commodities. Thus, effective screening of pesticide contamination in agricultural products and the environment is tremendously important to assure that pesticide remnants in commodities are under MRLs and the products are safe for consumption. Regular surveillance of pesticide dissemination in the environments is also a valuable way to control pesticide exposure. Pesticide residues are traditionally determined by chromatographic methods, including capillary electrophoresis (CE), liquid chromatography (LC) and gas chromatography (GC), coupled with either a conventional optical detection unit or a mass spectrometry (MS) system [1]. Chromatographic assay provides a great multiplex analysis of pesticides but its sensitivity and specificity require laborious and costly processes, expensive instrumentation, long turnaround time and highly trained personnel [2,3], which obviously limit the on-site and real-time monitoring of pesticide contamination. Colorimetric and antibody-based detection of pesticides have been developed to overcome the drawbacks of chromatography [4]. Currently, biosensor systems have also been a challenge in term of rapid, reliable and field-applicable detection of trace pesticides. Pesticides can trigger or inhibit enzymatic reactions relevant to their concentration, which can be monitored by colorimetric [5,6], fluorescent [4,7] or electrochemical [8] change. Pesticides can be complexed with their recognition molecules and deposit mass loads on quartz crystal microbalance (QCM) [9,10] or cause surface plasmon resonance (SPR) shift [11] corresponding to their concentration. Electrochemical biosensors are frequently applied for pesticide analysis, in which the pesticide interacts with its recognition elements (e.g., nanocomposite materials [12], acetylcholinesterase enzyme [8] or nanobodies [13]) and triggers redox activity changes to determine its concentration.

Recently, aptamers have gained much attention as recognition elements for diverse targets, including small molecules, proteins, living cells and tissue. Indeed, aptamer-based detection systems are a rising trend in analytical technology development. Aptamers are short single-stranded oligonucleotide sequences, either DNA or RNA, that can fold into specific conformations and promptly bind to cognate ligands with high avidity and specificity, making them suitable as effective recognition elements in diverse assay systems. More importantly, in addition to their antibody mimetic activity, aptamers also have several advantages over antibodies in terms of high thermostability, protease resistance, cost effectiveness in in vitro production, minimal batch–batch variation, small size, ease of modification and handling and target versatility [14,15]. Recently, aptamers have been successfully used in various promising applications, such as diagnostic, drug delivery, therapeutic and molecular imaging applications. Nonetheless, the selection of potential aptamer sequences for the specific recognition of small molecules, like pesticides, remains a challenge due to technical difficulty, especially at the partitioning process. In addition, pesticides have often been found in trace amounts of contamination and there are several hundreds of varieties. The discovery of aptamers and their development as biosensors for pesticide recognition is particularly interesting, as previously emphasized by Liu et al. [16] and Sekhon et al. [17]. With the rapid and continuing growth of aptamer selection and application for pesticide analysis, we need to keep an eye open for relevant updates and put effort to make them applicable and affordable in real practice. Thus, this paper reviews the progress of aptamer-based assays for pesticide determination, termed pesticide aptasensors, mainly focused on pesticide-specific aptamer selection, essential characterization and modification and utilization in numerous platforms to improve the efficacy of pesticide analysis and fulfill the ASSURED (affordable, sensitive, specific, user-friendly, rapid and robust, equipment-free and deliverable to end users) principle of biosensor development.

## 2. Generation of Aptamers against Pesticides

An aptamer requires either single-stranded DNA or RNA with a length of approximately 15–100 nucleotides to maintain its specific recognition and discrimination of the cognate target in a heterogeneous milieu [18]. Aptamers can also be found in the form of peptides [19]; however, that is beyond the scope of this review, which focuses on only nucleic acid aptamers, unless otherwise stated. Naturally, aptamers can be found in living organisms in the form of riboswitches, which function by binding with their ligands for subsequent regulation of specific gene expression [20]. Aptamers generated in vitro can be systematically isolated from a large pool of 10^12^–10^15^ synthetic oligo sequences using an iterative process called systematic evolution of ligands by exponential enrichment (SELEX). Since being first established in 1990, concurrently by Ellington and Szostak—who isolated dye-specific RNA [21]—and Tuerk and Gold—who developed T4 DNA polymerase-specific RNA [22]—the SELEX process has been applied and modified for the discovery of aptamers against a broad range of targets, such as proteins [23], metals [24], toxins [25], bacteria [26] and cancerous cells [27]. The details of the conventional SELEX procedure have been described elsewhere [28,29].

**Figure 1:**
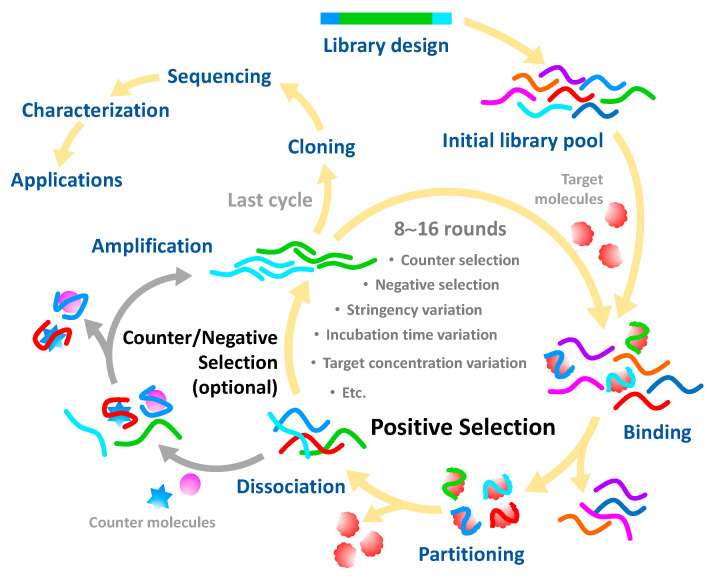
Typical steps of the systematic evolution of ligands by exponential enrichment (SELEX) procedure.

Briefly, SELEX involves the repetitive consecutive steps of binding, partitioning, dissociating and amplifying [30] (Figure 1). In general, each strand of an oligonucleotide library is composed of a random sequence flanked by two known sequences at its 5′ and 3′ termini for specific hybridization with primers during the amplification step. Through positive selection, oligonucleotide sequences are allowed to behave in a native tertiary structure for free recognition of the target, particularly by heating to 90–95 °C and cooling on ice for a period of time (5–10 min) and subsequently incubated with the target molecules. The target-bound oligonucleotides are partitioned from the unbound species and then amplified by polymerase chain reaction (PCR) to increase the copy numbers for execution of the subsequent rounds of the selection process. The successive processes of SELEX are usually carried out over about 8–15 cycles. After the final round of selection, the target-bound oligonucleotides are subjected to sequencing and some essential properties are characterized, such as affinity and selectivity for the target, molecular structure and potential uses. Over the past three decades, the SELEX process has been documented in more than 30 variants and systematically reviewed in many studies [31]; the variants are mainly intended to achieve some of the following: enhanced specificity and affinity, reduced processing time and complexity, expanded target prevalence and improved high-throughput screening.

**Figure 2:**
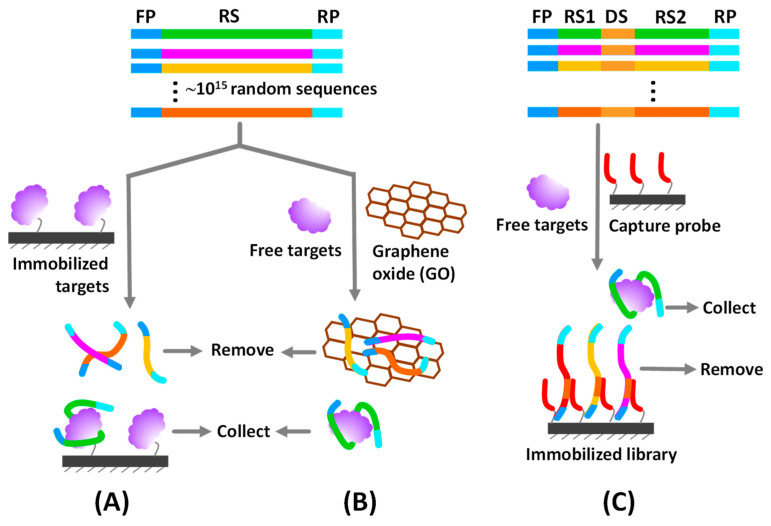
Schematic representation of common aptamer library designs and isolation concepts: (**A**) target immobilization, (**B**) immobilization-free and (**C**) library immobilization approaches (III). FP and RP, forward and reverse primer binding regions; RS, random sequence region, with RS1 and RS2 denoting random sequences at locations 1 and 2; DS, a docking sequence used for complementary binding with capture probe.

Since their invention, synthetic aptamers against pesticides have not been reported in SCOPUS until 2004, when Mulchandani released his discovery about an aptamer with a 10 µM affinity constant against the herbicide atrazine [32]. Over the 30 years of development, there are aptamers for only about 20 established pesticide targets (summarized in Table 1), even if numerous pesticides are currently problematic. There are some barriers impeding the discovery of aptamers against pesticides and other toxic agrochemicals. The first obstacle is that the SELEX process is time-consuming and requires skillful personnel. The second is that most pesticides have a small molecular structure, making their aptamer complexes difficult to partition from random oligonucleotide pools. Immobilization of either target molecules or random library sequences onto the solid phase is frequently carried out to circumvent this drawback (Figure 2A,C). However, in some circumstances, immobilization can be struggle with its own obstacles. For example, small target molecules like pesticides may lack functional groups to couple on the solid surface, while immobilization of the library sequences can interfere with the native configuration and hinder the binding ability of potential aptamers. Hence, the immobilization-free SELEX process offers an alternative choice for aptamer selection (Figure 2B). However, its technology currently requires the aptamer to undergo significant conformational changes upon duplexing with the cognate target for successful discrimination from the random sequences. Therefore, careful design of the SELEX procedure is a key success factor in aptamer selection. Cases of successful pesticide-specific aptamers are reviewed and conceptualized in the following subsection.

### 2.1. Immobilized SELEX Approach

Target-bound and unbound oligo sequences can be simply partitioned using a solid phase (e.g., chromatographic columns, microplates, microbeads, nanoparticles) modified with target molecules or random oligomers. The target-specific aptamer sequences can be selectively collected in their relevant stationary or liquid constitution for further characterization or execution of the next selection rounds.

#### 2.1.1. Immobilization of Target Molecules

Target immobilization SELEX is traditionally employed to isolate aptamers against various pesticides. For instance, N-fluoroacetyl glycine, a fluoroacetamide analog, was conjugated with bovine serum albumin (BSA) to serve as a molecular bridge for adsorption and the presentation of conjugated fluoroacetamide on a microplate. After incubation with a random ssDNA pool, the fluoroacetamide-specific aptamers could be isolated by washing out the free oligonucleotides and collecting the bound components for further characterization [47]. Likewise, an aptamer specific to the fungicide carbendazim was discovered, similar to the previously mentioned SELEX platform, which relies on carbendazim-BSA coated microplate [39]. Albumin plays a key role in immobilization of small molecular targets on the microplate and particulate surfaces because of its high adsorbability to the liquid/solid interface driven by electrostatic and hydrophobic interactions [53]. Importantly, based on its proteinous nature, albumin consists of many amino and carboxyl moieties, making it easy to conjugate with exogenous molecules for later display of specific recognition by the aptamer. Although conjugation with albumin provides uncountable benefits for small target immobilization, albumin may exert non-specificity and non-selectivity by interacting with the random pool sequences itself. Besides, albumin-conjugated target molecules may not be able to behave in their native configurations, resulting in misidentification of the most specific aptamers and lessening the potential utilization of selected aptamers.

However, unlike antibody production, there is no need to link the small target molecule with the hapten carrier to trigger aptamer production. In this regard, direct coupling of fipronil with carboxyl-modified magnetic beads was successfully demonstrated to isolate the aptamer with high affinity to the insecticide fipronil at a dissociation constant (*K_D_*) of 48 nM [46]. Moreover, the biotin–streptavidin system has been employed for target immobilization in the SELEX process, which helps to enhance the specificity and efficiency of immobilization and avoid molecular hindrance, as can happen in the target–albumin conjugation system. Williams et al. reported their success in developing aptamers against atrazine [35] and bromacil [38]. Derivatives of atrazine and bromacil were covalently coupled with biotin and later specifically immobilized to streptavidin-coated magnetic particles. The immobilized atrazine and bromacil were employed to fish out their specific aptamers in the heterogeneous ssDNA sequences with the assistance of magnetic separation after a certain period of incubation. Magnetic nanoparticles are robust and able to decorate with a variety of functional groups that are required for the appropriate coupling of target molecules. Due to its paramagnetic property, aptamer bound with target-modified magnetic beads can be easily collected by pulling down with the permanent magnet, thus making the SELEX process more convenient, cost-effective, time-saving and applicable in a small volume setup.

#### 2.1.2. Immobilization of Random Library

Some pesticides and other small molecules do not have functional groups or their derivatives to use for conjugation with the carrier protein or immobilization on the solid surface. Hence, immobilization of the library sequences in place of the target molecules is an option to overcome such limitation. Basically, each sequence of a library pool is specially designed to contain a docking region that can be hybridized with the capture probe immobilized on the solid supporter. A docking sequence is inserted either between two random regions [33] or at the terminal region [36] of the library sequence (Figure 2C). This SELEX variant is commonly called capture-SELEX. Its working principle is undeniably dependent on the structure-switching property of the aptamer upon binding with the specific ligand. The captured aptamer transforms its conformation to fit with ligand structure, which triggers the release and partitioning out of the aptamer from the capture probe, where the ligand-specific sequences can be collected as unbound constituents. Furthermore, library sequences can be immobilized on the solid phase without the docking region. For example, acetamiprid aptamer, a well-known pesticide aptamer, was developed by PCR amplifying the ssDNA library with biotin-labeled primer and afterward the product was immobilized on agarose resins via the biotin-streptavidin linkage. The anti-sense strand served as the capture probe used to construct the library sequences on the solid support. Incubation of acetamiprid with the immobilized ssDNA caused the release of acetamiprid-bound sequences into solution, which were collected for enrichment and characterization after the ssDNA-immobilized resins were discarded [33].

The capture-SELEX method facilitates the discovery of aptamers for the recognition of multiple targets, which is a common limitation of the target immobilization approach. Thus, a broad-spectrum aptamer was successfully developed to target four organophosphorus insecticides—phorate, profenofos, isocarbophos and omethoate [51]. It is important to keep in mind that capture-SELEX is restricted by the structure-switching ability of the aptamer; some aptamers do not significantly undergo conformational changes to detach from the capture probe, leading to misidentification of potential sequences. Furthermore, the capture sequence itself can interfere with the 3D configuration and get involved with the target-recognition capability of the aptamer as well.

### 2.2. Non-Immobilized SELEX Approach

#### 2.2.1. Capillary Electrophoresis-Based SELEX Process (CE-SELEX)

Capillary electrophoresis (CE) can be integrated effectively with the SELEX process for the isolation of target-bound aptamers from a heterogeneous oligonucleotide library without the need for the immobilization step. Binding of aptamer with the cognate ligand results in conformational change and molecular mass shift, which can be fractionated from the unbound library by means of electrophoretic mobility differences in a microfluidic channel of the CE apparatus. CE-SELEX allows ssDNA library sequences and target molecules to interact freely in their native configurations under an aqueous environment. It helps to reduce the SELEX cycles by about 2–3 times, which requires only 2–6 cycles and shortens the turnaround time from a few weeks to a few days to catch the high-affinity aptamer compared to the conventional SELEX [54]. CE-SELEX was employed to select the first recorded pesticide aptamer against atrazine, which took just 6 repetitive cycles to obtain an aptamer consisting of 40 randomized oligomers with a dissociation constant of 890 nM [37]. Even if CE-SELEX can cut down the processing time of the selection procedure, it requires costly and sophisticated CE facilities, which may not be available in many laboratory settings. Also, CE-SELEX may not effectively isolate aptamer for some small molecules, because CE may be unable to discriminate small electrophoretic mobility differences between target-bound and unbound sequences [28].

#### 2.2.2. Physical Adsorption-Based SELEX Process

This SELEX variant relies on the nonspecific adsorption of randomized library strands onto the surface of nanomaterials (i.e., graphene oxide or gold nanoparticles). Once it binds with the target molecule, the target-specific aptamer sequence is detached out of the surface and can be collected for further elucidation. It has been found that ssDNA simultaneously adsorbs on graphene oxide (GO) sheet via hydrogen bonding together with π–π stacking mechanisms and its affinity is much stronger than the dsDNA’s [55]. Considering this fascinating phenomenon, GO has been adopted in the partitioning step of the SELEX process to avoid the tricky chemical immobilization of target and library.

Target-specific aptamers are desorbed from the GO sheets due to their conformational changes upon refolding to complex with specific ligands. Supernatant containing target-ssDNA complexes can be collected using simple centrifugation to remove GO substrate bound with nonspecific library sequences [56]. GO-SELEX is convenient and helps to shorten the turnaround time of the aptamer selection process, which takes about 240 min per consecutive round. Besides screening the aptamer for a single target, GO-SELEX was successfully deployed for multiplex isolation of aptamers against three pesticides: tebuconazole, mefenacet and inabenfide. The obtained aptamers displayed dissociation constant values at the nanomolar level [57]. The same group extended the GO-SELEX method to develop aptamers against two pesticides, iprobenfos and edifenphos, with dissociation constant values of 1.67 and 0.038 µM, respectively [50]. GO-SELEX provides simplicity, high speed and high-throughput, which are advantages in facilitating the advancement of aptamer selection and utilization. More recently, besides detection, the usefulness of gold nanoparticles (AuNPs) in aptamer selection was demonstrated by Chatterjee and co-workers [45]. In this approach, termed GOLD SELEX, AuNPs serve as a solid surface to adsorb and partition the randomized ssDNA library from the target-specific aptamer, under a similar paradigm as GO-SELEX. Dichlorvos-specific aptamers were successfully fished out by this novel approach with *K_D_* at the submicromolar level.

## 3. Improvement of Aptamer Specificity and Affinity

Aptamers have limited chemical diversity since they are constructed from a combination of only four nucleobases to produce certain conformations and binding motifs for specific recognition of target molecules. In contrast to the 20 amino acid compositions with their post-translational modifications, antibodies can generate relatively more variations of binding pockets, increasing the possibility to selectively and strongly recognize target molecules than the equivalent oligonucleotide aptamers. Hence, to increase the possibility of acquiring high-affinity and selective aptamers against targets, many strategies have been employed, including but not limited to negative selection, counter selection, stringency amendment, aptamer length optimization and post-selection modification.

### 3.1. Eliminating Non-Specificity with Selection Matrix and Counter Molecules

The SELEX process based on the target-immobilization approach can provide nonspecific sequences that bind undesirably with the immobilizing matrix, such as carrier proteins, linker chemicals, magnetic beads and microplate surfaces. Therefore, the so-called negative selection process is applied after a few cycles of positive selection toward the target in order to eliminate such nonspecific sequences, in which the immobilizing matrix without target is incubated with the enriched library pool. Subsequently, matrix-bound sequences are discarded to collect the unbound constituents for successive rounds of positive selection [58]. In addition, the specificity of aptamers can be enhanced by challenging the library pool with counter selection, which is deliberately similar to negative selection but utilizes structurally similar target molecules (e.g., metabolites and derivatives of target molecules) instead of the matrix substrate to shed non-specific oligo sequences [59]. Negative selection and counter selection have been used interchangeably in many studies. Generally, positive, negative and counter selection can be alternately and repeatedly performed throughout the SELEX process to achieve the most effective aptamers with high affinity and selectivity toward target molecules.

### 3.2. Enhancing Affinity with Stringency Modification

Aptamers specifically recognize their ligands by means of conformational fit and intermolecular forces, such as hydrogen bonding, electrostatic forces, van der Waals interaction and so forth. The negatively charged nature of oligonucleotide aptamers can make them nonspecifically bind to cationic substances, causing undesirable performance. To fine-tune the affinity, high stringency conditions are often applied to the SELEX process, such as lowering the concentrations of library and target molecules, decreasing the incubation time, adding denaturing detergents and chelators and varying the salt gradients. These strategies can be applied in positive selection as well as counter and negative selection to eradicate inappropriate binding events between aptamers and targets or nonspecific species.

### 3.3. Trimming off Nonessential Sequences for Target Recognition

In general, full-length aptamers isolated by the SELEX process consist of 60–100 oligomers, in which about 25–50 nucleotides belong to the random region and the remaining sequences at both termini are involved in hybridization with forward and reverse primers (~20 nucleotides each) [60]. The primer binding regions are considered as unimportant parts of the target-binding domain that can, in turn, exert a nonspecific interaction or hinder a randomized region from the target molecules. Truncation of nonessential binding components of aptamers has been proven to enhance affinity, specificity, stability and cost-effectiveness [61]. For example, truncated sequences of acetamiprid-specific aptamer (25, 37, 40 and 43 oligomers) were developed by sequentially deleting the parental aptamer (49 nucleotides) from the 5′-end, where the stem loop was predicted to be absent [62]. By integrating with AuNPs, the truncated aptamers provided higher detection sensitivity (1.8–3.3 fold) to acetamiprid than the parental aptamer, in which its excess sequences outside the binding region might nonspecifically interact with AuNPs and diminish the colorimetric response of the detection system.

### 3.4. Understanding Molecular Interaction for Rational Design and Selection

Current advances in computational power and algorithms provide an in-depth understanding of the molecular interactions between recognition elements and ligands, which can be applied to improve the selection process and enhance the recognition performance of aptamers. The 3D-configuration of aptamers can be computationally predicted and used as a template for docking with potential ligands. Molecular docking not only provides binding energy but also explains the interaction environment between aptamers and ligands at static condition. In addition, physical movement and stability of the aptamer–ligand complex can be scrutinized by molecular dynamic (MD) simulation. For instance, a potential aptamer for diazinon was virtually re-screened from a set of 12 original aptamers [43]. A molecular docking study suggested that an aptamer coded as DF20 had the most potential for diazinon recognition because of its low inhibitory constant (*K_i_*), high binding energy and low root-mean-square deviation (RMSD) compared to other aptamer sequences. MD simulation also demonstrated a stable DF20-diazinon complex [44]. Recently, the DF20 sequence has been widely utilized to develop diazinon aptasensors based on colorimetric [44], fluorescence resonance energy transfer (FRET) [63] and electrochemistry [64] platforms.

Belinskaia et al. demonstrated the rational design of paraoxon aptamer using computational simulations and in silico mutation of previously published aptamers for multiple organophosphorus pesticides. The most probable binding site for paraoxon on a 3D-aptamer structure was identified and then its surrounding oligonucleotides were virtually mutated to improve recognition efficacy. Among 35 nucleotide residues of organophosphorus aptamers, nucleotides T17, C18, T19 and T20 were proposed to play a major role in paraoxon recognition. Rational modification was conducted by virtual substitution of such oligomers with three other nucleobase variants. Free binding energy analysis suggested that T17C and T17C-C18T mutated sequences were the most effective paraoxon-binding aptamers with an estimated *K_D_* value around 1 µM, which was comparable to the previous report on other organophosphorus targets (0.83 and 2.5 µM) [65].

## 4. Essential Characterization of Pesticide Aptamers

Pesticides are much smaller in size than aptamers and reveal very little about their interaction events, making it difficult not only to isolate them but also to characterize their essential properties. Characterization is required to point out the general properties (e.g., secondary and tertiary conformations) and ligand binding performance (e.g., affinity and selectivity) of aptamers, which are important in choosing the right aptamer for the right applications.

Computational analysis is a useful method to illuminate the conformation of the candidate aptamer. The Mfold web server is typically employed to elucidate the secondary structure and relevant Gibbs free energy value (∆G°) of aptamers [33,35,50]. Abraham et al. determined a candidate atrazine-specific aptamer (R12.4) folding by using the Mfold service and found that its structure belonged mostly to a random region and partially to constant regions at the 5′ and 3′ termini. Guided by the predicted structure, truncation of the R12.4 aptamer was initiated by trimming the oligo sequences in both constant and random regions located outside the major hairpin structure in order to reduce cost and increase the efficiency of aptamer synthesis [36].

Furthermore, the secondary structure of aptamers can be experimentally elucidated by circular dichroism (CD) spectroscopy. Conformational variants of aptamers can be distinguished by absorption characteristics of left- and right-polarized light, displayed as unique negative and positive bands of the CD spectrum. For example, a B-form DNA structure shows a negative and a positive band at around 245 and 270 nm, respectively [36]. G-quadruplex structure usually shows a maximum peak at ~260 and a minimum peak at ~240 nm [66]. In addition to its use in exploring the secondary structure, CD is also a valuable tool for revealing the structural changes of aptamers upon binding with ligands [67]. However, the CD spectrum represents only global features of molecules; it cannot provide deep speculation into the binding pocket, which needs to be interpreted with other means, such as computational structure prediction, nuclear magnetic resonance (NMR) or X-ray diffraction, to gain more detailed structural information of the aptamer-ligand complex [66,68,69].

The aptamer-target affinity is generally determined in terms of the binding constant (*K_A_*) or its reciprocal dissociation constant (*K_D_*), which can be estimated from the apparent isotherm of aptamer and ligand titration derived by an analytical method such as surface plasmon resonance (SPR), isothermal titration calorimetry (ITC), fluorescence saturation binding assay or capillary electrophoresis (CE). The fluorescence binding assay is simple and requires less sophisticated instruments but needs to label aptamers with fluorescent probes. Commonly, the target molecule is immobilized and incubated with various concentrations of fluorescent-labeled aptamer. After washing out the unbound sequences, the target-bound aptamer is eluted and fluorescence intensity is measured to determine the *K_D_* parameter based on an appropriate fitting model of the binding isotherm, such as nonlinear regression analysis [35,46]. In order to circumvent the target immobilization step and avoid aptamer alteration by the labeled fluorophore, molecular beacons can be used as fluorescent probes to generate the binding curve of the aptamer–target complex [51]. This technique allows aptamers to freely interact with targets in their native conformation and lets beacon probes hybridize with free aptamers, which changes the fluorescence emission relative to the complex fraction.

Recently, SPR and ITC have been widely adopted as label-free methods for analyzing biomolecular interactions. SPR functions on the principle that the binding of aptamers with immobilized targets or vice versa, on an SPR chip causes refractive index changes, traced in real time as a sensorgram. SPR provides crucial binding parameters, especially *K_A_*, *K_D_*, kinetic rate constants (*k_on_* and *k_off_*) and stoichiometry by estimating from the aptamer–ligand binding isotherm [47,70,71]. ITC directly measures temperature change as a result of the aptamer and target interaction. It elucidates the essential information about thermodynamic parameters of the molecular interaction, such as Gibbs free energy change (∆G°), entropy change (∆S°) and enthalpy change (∆H°), which helps explain the interaction mechanism, major binding forces and spontaneity of the aptamer–ligand complex. Both SPR and ITC provide kinetic information about the interaction mechanism with the requirement of small sample volume, making them applicable for characterization of the aptamer–pesticide duplex [36,47,50,57].

## 5. Current Advances in Aptasensors for Pesticide Determination

Aptamers play an important role in the effective recognition of many biosensor systems since they have high affinity and specificity for targets and integrate easily with a variety of available signal transduction platforms. Over the past few decades, certain aptasensors for pesticide analysis have been developed, mainly relying on colorimetric, fluorometric and electrochemical assays. The use of various systems such surface-enhanced Raman scattering (SERS) and microcantilever techniques has been observed occasionally. The working principles, merits and boundaries of pesticide aptasensor development are systematically reviewed and emphasized herein.

### 5.1. Colorimetric Pesticide Aptasensors Based on Nanoparticle Agglomeration

Noble metal nanoparticles (NPs) have a unique localized surface plasmon resonance (LSPR) property, in which a collective oscillation of electrons at the NPs’ surface resonates with and selectively absorbs a certain relevant frequency of incident light. This phenomenon is confined to the nanometer scale, comparable to the wavelength of light, rather than occurring along the entire metallic–dielectric interface [72,73]. LSPR creates a strong absorption or scattering peak, producing a distinctive color of NPs that can undergo shade alteration as a result of surface dielectric changes caused by many factors, such as size, shape and interparticle distance variations [74]. NPs have been actively applied in the development of highly sensitive biosensors owing to several advantages, especially enable naked-eye interpretation, high sensitivity for quantitative measurement, high robustness, simple synthesis, cost-effectiveness and ease of portability [75]. Gold nanoparticles (AuNPs) and silver nanoparticles (AgNPs) are among the top NPs applied as indicator in a variety of detection platforms, such as lateral flow dipstick, electrochemical and SERS formats, for chemical, biological and medical uses [76].

Dispersed AuNPs typically exhibit a red-pink color that can be turned to purple-blue after agglomeration. This color transformation can be simply quantified using absorbance measurement. AuNPs in dispersion generally absorb light in a wavelength between 400 and 550 nm, with a maximum peak at ~520 nm wavelength, which can be dramatically decreased and shifted to the red, ranging from 600–700 nm with a typical peak at ~650 nm after agglomeration. Therefore, the absorption ratio of A650/A520 has been routinely used to monitor the degree of AuNP aggregation. AgNPs exhibit different colors, mainly displaying yellow or light brown (400–420 nm absorption peaks) in the dispersion form and turning to orange or dark brown with a dramatic decrease of absorbance in the aggregated form [77]. Generally, NPs are stabilized in uniform dispersion by coating with negatively or positively charged molecules to provide strong electrostatic repulsion against van der Waals attraction and this type of stabilization is known as the electrostatic mechanism [78]. Besides this, NPs can also be stabilized by steric effects among the functional moieties on the NPs’ surface and this mechanism is referred to as steric stabilization. A combination of both mechanisms causes electrosteric stabilization [79]. Many biological and chemical entities can modify the stability and trigger the dispersion-aggregation transformation of NPs, suggesting a promising role of NPs in the rising trend of colorimetric assay development.

**Figure 3:**
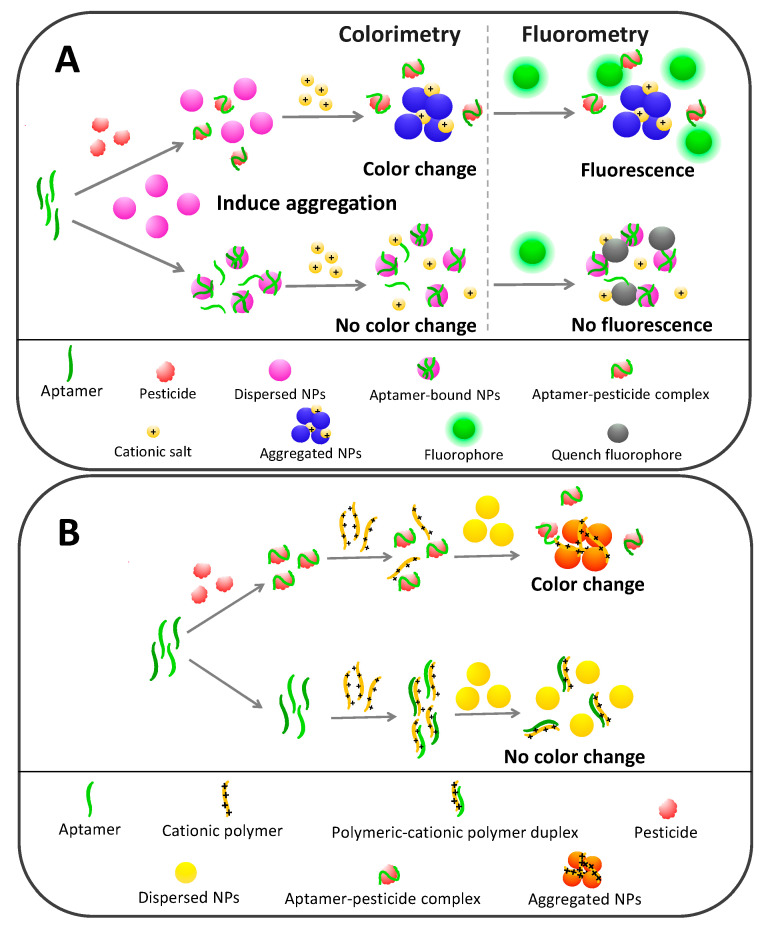
Schematic representation of general concept of nanoparticle-based colorimetric aptasensors for pesticide detection. (**A**) Color production is realized by salt-induced nanoparticle (NP) aggregation, which can be further extended to a fluorometric system by NP-induced fluorescence quenching. (**B**) Important role of cationic polymers in triggering NP aggregation in colorimetric pesticide aptasensors.

Aptamer-based biosensors for pesticide detection rely mostly on AuNPs and a minority on AgNPs to serve as color indicators. There are at least three key success factors for developing nanoparticle-based colorimetric aptasensors toward pesticides: (1) the conformational changes and dislocation properties of aptamers upon binding with targets, (2) the stabilization activity of aptamers toward NPs and (3) the color change phenomenon of NPs. As schematically depicted in Figure 3A, in the absence of pesticide targets, aptamers act as a stabilizer to prevent NP aggregation by the electrosteric mechanism. Their single-stranded nature makes aptamers behave in a random coil structure, enabling their exposed nucleobases to interact and attach on the surface of NPs. ssDNA has a higher potential to stick to AuNPs than double-stranded DNA [80,81].

Aptamer-bound NPs favorably stay in dispersion and resist aggregation because negatively charged phosphate backbones of bound aptamers exert more repulsion power than the van der Waals attractive force. Conversely, pesticide targets specifically bind to aptamers and induce conformational changes to form supramolecular aptamer–pesticide complexes, which are relatively rigid and unlikely to stick to the NPs, leaving them destabilized and prone to aggregation [67]. With the essence of the designable control of NP aggregation or disaggregation being to display a dramatic color change in response to the analyte concentration, NPs have many advantages for analytical purposes.

Two approaches have been utilized to provoke AuNP or AgNP aggregation in aptamer-based pesticide detection systems: salt-induced aggregation (e.g., high Na^+^ concentration) and cationic polymer-induced aggregation (e.g., lysine/arginine rich peptide [82] and poly-diallyldimethylammonium chloride [83]). In the presence of pesticide targets, cationic charges belonging to either of those inducers disrupt the electrostatic equilibrium of the surface of NPs [84], which consequently undergo aggregation and color conversion corresponding to the pesticide concentration. On the other hand, in the absence of targets, NPs remain stabilized by aptamers and resist agglomeration in the positively charged milieu, resulting in no color change. It is noted that each sequential step of colorimetric aptamer-based detection can be interchangeable as observed in many studies.

Unlike salt-induced NP aggregation, cationic polymers do not provoke aggregation only by charge destabilization but also by crosslinking among the NPs (Figure 3B). More importantly, in the absence of pesticide targets, cationic polymers form duplexes with aptamers and lose their agglutination activity toward NPs. In contrast, pesticide targets have a higher capacity to competitively bind with aptamers than cationic polymers, which leaves cationic polymers free to aggregate NPs and generate colors in correspondence with the pesticide quantity.

AuNP- and AgNP-based colorimetric aptasensors show excellent performance in pesticide determination, in which the limit of detection (LOD) at the picomolar to nanomolar level has been demonstrated in various sample matrices, including soil, water, vegetables, fruits and plasma specimens, as summarized in Table 2. For example, simple aptamer-based sensing of acetamiprid in soil was developed by utilizing the 20-mer acetamiprid-specific aptamer as a recognition element together with the citrate-capped AuNPs as a color indicator. A detection range between 75 nM and 7.5 µM with a detection limit (LOD) of 5 nM could be achieved upon adding high concentration (500 mM) NaCl to initiate the corresponding color intensity, as exemplified in Figure 3A [67]. A similar principle was employed for multiplex determination of iprobenfos and edifenphos. Dual target aptamers were developed based on the GO-SELEX procedure, as mentioned above and later integrated with the NaCl-induced AuNPs aggregation system, which was able to monitor iprobenfos and edifenphos concentrations down to 10 and 5 nM, respectively [50].

Bala et al. demonstrated ultrasensitive malathion aptasensors based on cationic polymer-activated AgNP agglomeration. The yellowish AgNPs turned orange in the presence of malathion as a consequence of aptamer–malathion complex formation, triggering free hexapeptide (KKKRRR)-induced AgNP aggregation. This system provided a very low LOD at 0.5 pM and a linear response ranging from 0.01–0.75 nM of malathion [85]. Furthermore, superb LOD for malathion was enhanced about 10 times (0.06 pM) by assembling malathion-specific aptamers and AuNPs with polydiallyldimethylammonium (PDDA) polymer as a colorimetric nanoprobe. This platform also provided a broader linear detection range (0.5–1000 pM) compared to the AgNP and cationic hexapeptide system [86]. Both AuNPs and AgNPs have been proven to provide excellent performance for pesticide analysis of aptasensor platforms. However, AuNPs have gained more attention in colorimetric assays than AgNPs, although they are more expensive and have lower extinction coefficient values at equivalent particle sizes. This is because AuNPs are stable and their optical properties are highly dependent on the interparticle distance, making them sensitive and advantageous in analytical applications [87]. Nonetheless, in some aptamer-based sensing systems, AuNPs may resist salt-induced aggregation and not produce a color change, because aptamers may not undergo conformational alteration and remain stabilized in the dispersed AuNPs even if in the presence of target molecules [36,88]. The detection performances of colorimetric pesticide aptasensors are summarized in Table 2.

### 5.2. Fluorometric Aptasensors for Sensitive Agrochemical Detection

Fluorescence assay has been widely adopted in analytical systems for several decades and is still a growing trend. The fluorescence technique offers several advantages, including but not limited to high sensitivity and specificity due to its impressive signal-to-noise (*S*/*N*) ratio and specific excitation and emission wavelengths, ease of interpretation either by the naked eye or transducers, rapid results and field applications. In aptamer-based pesticide detection, fluorescence can play a major role in both signal-on and signal-off sensing platforms, based on different signal generation strategies, such as fluorescent probe labelling, fluorescent probe hybridization, fluorescence quenching and fluorescence resonance energy transfer (FRET). Detection systems of many designs have been found, which may be as simple as one-pot detection or require the complex immobilization and separation processes. The integration of fluorescent active materials and nanocomposites has been used frequently for the development of pesticide aptasensors, in particular rhodamine, fluorescein, quantum dots (QDs), carbon dots (CDs), upconversion nanoparticles (UCNPs), GOs and AuNPs, as illustrated in the following sections, and their performance is summarized in Table 3.

#### 5.2.1. Dual Colorimetric and Fluorometric Aptasensors Based on Noble Metal Nanoparticles

The plasmonic property of AuNPs and AgNPs is useful not only for color production but also for fluorescence conversion, which helps promote the development of a dual colorimetric and fluorometric aptasensor platform. This technique is a kind of extension format of colorimetric aptasensors based on NP aggregation as previously mentioned. For instance, dispersed AuNPs effectively quench the fluorescence emission of many fluorescent materials as a consequence of resonance energy transfer (RET) [109] and/or the inner filter effect (IFE) [110]. This phenomenon is important in aptasensor development because in the absence of target pesticides, aptamers remain wrapped around and stabilize the AuNPs in dispersion to exert a quenching effect on the fluorescent probes, so that neither color changes nor fluorescence emission is observed (Figure 3A). On the other hand, binding of aptamers with pesticides leads to aggregation of free AuNPs and less fluorescent quenching effect, producing a dual-readout signal in terms of the colorimetric change and fluorescence emission consistent with the target concentration.

Wang et al. developed a dual-mode aptasensor for monitoring of isocarbophos using a combination of AuNPs and Zn_2_GeO_4_:Mn persistent luminescence nanorods (PLNRs) to produce color changes and fluorescence emission in response to the isocarbophos in vegetable samples. PLNRs showed a maximum emission peak at 537 nm, which was strongly absorbed by the dispersed AuNPs; otherwise they remained stable and emitted fluorescence in the aggregated AuNP milieu. The colorimetric mode, based on the absorptivity of aggregated AuNPs, provided an LOD of isocarbophos of 7.1 µg/L, while about 10 times better LOD (0.54 µg/L) was provided by the PLNR fluorometric partner [91]. As another example, AuNPs and NH_2_-NaYF_4_:Yb,Ho@SiO_2_ upconversion nanoparticles (UPCNPs) have been applied as colorifluorometric indicators for acetamiprid aptasensor [96]. UPCNPs emit maximum fluorescence at 545 nm, which largely overlaps with the absorption band of AuNPs. In the absence of acetamiprid, the aptamer-bound AuNPs resisted salt-induced aggregation but electrostatically adsorbed on the UPCNP surface and met the FRET criteria. Fluorescence of UPCNPs was quenched by AuNPs serving as acceptors that acquired energy and caused fluorescence loss of the excited UPCNPs. Once the aptamers unbound from the AuNPs to specifically capture the acetamiprid target, AuNPs underwent aggregation and recovered the fluorescence emission of UPCNPs with the extent of acetamiprid concentration. Both color changes and fluorescence emission could be observed by either the naked eye or machinery, with ~3.2 nM of LOD. Based on a similar principle, relatively low LOD for chlorpyrifos (3.8 nM) and carbendazim (2.33 nM) was achieved by coupling AuNPs with a terbium-based metal-organic framework (Tb-MOF) [99] and rhodamine [97], respectively.

#### 5.2.2. Graphene Oxide- and Carbon Nanomaterial-Based Fluorescence Aptasensors

Carbon-based nanomaterials, especially graphene oxide (GO) and carbon nanotubes (CNTs), have gained much interest in analytical applications. GO is a single-layer hexagonal carbon sheet that is strongly oxidized to bear oxygen-containing groups, for example, hydroxyl, epoxy and carboxyl groups [111]. CNTs are made up of a hexagonal carbon sheet material but they fold into a tubular structure in either single-wall (SWCNT) or multi-wall (MWCNT) format. Both GO and CNTs have an sp^2^ carbon nanostructure, which has a large surface area to strongly and easily interact with other molecules, like aptamers, via π–π stacking and van der Waal force, as aforementioned. The sp^2^ hybrid carbon network also promotes GO and CNTs to be good electron transfer materials and strong fluorescence quenchers, making them very useful in electrochemical and fluorometric biosensors. GO and CNTs have been found to strongly quench the fluorescence of many materials (e.g., organic fluorophores, metallic quantum dots and carbon nanodots) through FRET or photoinduced electron transfer (PET) mechanism, in which they act as acceptors to take energy or electrons from donor fluorophores [111,112].

**Figure 4:**
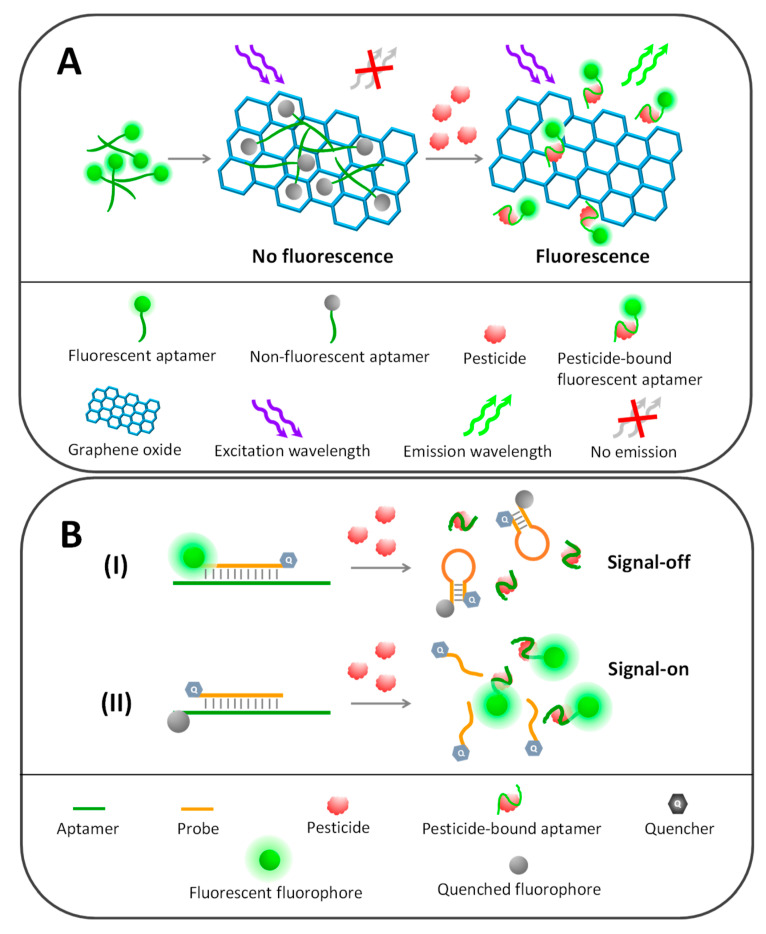
Schematic representation of fluorometric aptasensors for pesticide detection. (**A**) Fluorescence quenching and aptamer binding properties of graphene oxide and related carbon nanomaterials. (**B**) Fluorescent aptasensors using complementary probes: dual-labelled (I) and single-labelled (II) fluorescent probe.

As schematically depicted in Figure 4A, graphene-based fluorescent detection of profenofos has been developed using rhodamine-labelled aptamers as sensing probes. In the absence of profenofos, rhodamine-labelled aptamers favorably adsorbed on polyethylene glycol-functionalized GOs (PEG-GOs) and lost their fluorescence signal. The addition of target profenofos regained the fluorescence emission of rhodamine-labelled aptamers because their conformational change separated the labelled rhodamines from the GO quencher, providing a linear detection range of 0.5–100 ng/mL with an LOD of 0.21 ng/mL [103].

Besides organic fluorescent probes, metallic quantum dots have been widely adopted along with GOs to serve as promising sensing probes in pesticide aptasensors. For example, Zhang et al. developed an ultrasensitive omethoate aptasensor based on the combination of graphene quantum dot-conjugated aptamers (GQD-aptamers) and GOs. The fluorescence of GQD-aptamers could be turned on and off in the absence and presence of omethoate, respectively. This system could detect trace amount of omethoate at an LOD of 0.029 pM by means of fluorescence polarization analysis [104]. Moreover, the fluorescence quenching activity of carbon nanomaterials also influences the G-quadruplex–porphyrin complex. Li et al. established an isocarbophos aptasensor by splitting the isocarbophos-specific aptamer into two pieces, each of which carried a guanine-rich sequence known as the G-quadruplex, to serve as fluorescent reporters. Without the isocarbophos, both aptamer pieces randomly adhered onto the MWCNTs, diminishing the fluorescence of G-quadruplex as a consequence of energy transfer to the MWCNTs. In contrast, both aptamer pieces underwent conformational changes and stripped out of the MWCNTs to reunify and bind with the isocarbophos in the sample, consequently recovering the G-quadruplex’s fluorescence proportional to the isocarbophos concentration [101]. Alternatively, carbon nanomaterials have been proven to express intrinsic peroxidase-like activity [113], which can be retarded when interacted with aptamers, providing a benefit for aptasensor development. Nitrogen-doped carbon dots (N-CDs) were used together with aptamers for the detection of isocarbophos. As mentioned, aptamers preferably bind onto N-CDs and inhibit their catalytic activity, whereas isocarbophos produces a contrary effect by complexing with aptamers and leaving N-CDs free to catalyze the H_2_O_2_/TMB substrate and produce fluorescence signals according to the isocarbophos concentration. It could detect trace amounts of isocarbophos down to 0.015 µg/L with a linear range of 0.025–1.5 µg/L [92].

#### 5.2.3. Complementary Fluorescent Probe-Based Aptasensors

The oligonucleotide nature of aptamers enables them to hybridize with complementary oligo sequences, termed probes, which is very useful in analytical systems such as real-time PCR, loop-mediated isothermal amplification (LAMP), microarray and aptamer-based detection platforms. The successful application of complementary probes in analytical systems greatly relies on the energy transfer phenomenon of the conjugated fluorophore and the quencher.

Two main types of complementary fluorescent probes have been found in aptamer-based pesticide detection. The first is a dual-labelled probe, in which both termini of the probe sequence are tagged with each fluorophore and quencher pair (Figure 4(BI)). In addition to its ability to hybridize with the aptamer, the probe sequence was designed to undergo self-hybridization and form a hairpin structure (molecular beacon) that brings fluorophore and quencher close to each other and causes fluorescence quenching. On the other hand, if the dual-labelled fluorescent probe complementarily hybridizes with its target aptamer, the labelled fluorophore and quencher are separated, diminishing the quenching activity and resulting in fluorescence recovery of the labelled fluorophore. However, in the presence of the pesticide target, the fluorescence signal is turned off due to aptamer–pesticide complexation shedding the fluorescent probes to reform the hairpin structure and provoke fluorescence quenching.

In the second variant of fluorescent probes in pesticide aptasensors, fluorophore and quencher are separately conjugated with probes and aptamers, classified herein as the single-labelled fluorescent probe strategy (Figure 4(BII)). The hairpin structure of probe sequences is not necessary in this strategy but the labelled fluorophore and quencher pairs need to be in close proximity after the hybridization of probes with aptamers. So, careful design of the probe localization and the fluorophore labelling site on the aptamer sequences is key. The specific binding of pesticides with aptamers causes complementary probe release from aptamers and separates the fluorophore from the quencher, producing fluorescence signals corresponding to the pesticide concentration.

For example, Zhang et al. established aptasensors for multiplex detection of four organophosphates based on fluorescence polarization analysis using broad-spectrum aptamers and molecular beacon probes as sensing elements. Short hairpin oligonucleotide sequences of a molecular beacon were tagged with fluorescein as donor and DABCYL as quencher. No fluorescence emission was observed without the hybridization of molecular beacon with aptamers, where molecular beacon remained in the hairpin structure and brought about fluorescence quenching of fluorescein by DABCYL. This event could be observed in the presence of organophosphates that competitively bound to aptamers and released the molecular beacon to stay in fluorescence turn-off mode. The limit of quantification (LOQ) toward phorate, profenofos, isocarbophos and omethoate was found to be 19.2, 13.4, 17.2 and 23.4 nM, respectively [52].

Additionally, Dou et al. developed a nanoparticulate form of fluorescent beacon probes to monitor organophosphate insecticides. The fluorescein-labelled probes were decorated onto the AuNPs to serve as nanobeacon probes for isocarbophos, profenofos, phorate and omethoate. AuNPs not only served as nanocarriers but also quenchers toward the labelled fluorescein when organophosphates competitively bound to the aptamers and left immobilized beacon probes to undergo self-hybridization and turn off fluorescence emission. This system provided micromolar LOD values in the range of 0.035–2.35 µM toward those four organophosphates [108]. Recently, an aptamer-based fluorescent assay was established for the detection of fibronil in eggs. The fibronil aptamer was labelled with fluorescein, in which it was later captured and quenched by carboxy-tetramethyl rhodamine tagged complementary cDNA probes. The aptamer underwent conformational changes to bind with fibronil present in egg samples and released the quencher probe, resulting in fluorescence recovery corresponding to the fibronil level in the range of 25–300 ppb and LOD of 53.8 ppb [100].

#### 5.2.4. Solid Phase-Based Fluorometric Aptasensors

**Figure 5:**
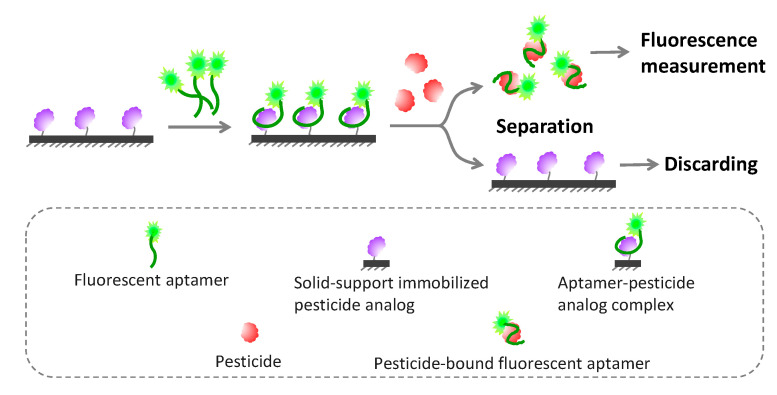
Schematic depiction of working principle of fluorescent pesticide aptasensors based on solid-phase immobilization.

The FRET-based assay may have some limitations, because it requires spectral overlapping and close proximity among FRET pair fluorophores, which may not be possible in some circumstances or may provide only a low signal-to-noise ratio readout. Solid phase fluorescence detection can overcome such limitations, in which target-bound aptamers are separated from unbound aptamers and then monitored. Normally, an aptamer is conjugated with a fluorescent dye and later captured on the solid supporter via an immobilized capture probe (Figure 5). Upon binding with the target molecule, the captured fluorescent aptamer transforms its conformation and dismisses out of the solid supporter, which can be collected and have its fluorescence intensity further measured related to the amount of target. Capture probes can be found in various formats, including as short oligonucleotides complementary to aptamers, as analogs of target molecules and as a polymer with the opposite charge to aptamers.

Recently, fluorescent aptamer-immobilized magnetic nanoparticles were fabricated for multiplex monitoring of pesticides (trichlorfon, glyphosate and malathion). The supernatant containing pesticide-bound fluorescent aptamers could be collected and fluorescence intensity could be measured after simply discarding the magnetic beads bearing unbound aptamers, which provided LOD for trichlorfon, glyphosate and malathion at 72.20, 88.80 and 195.37 ng/L, respectively [106]. Instead of using complementary DNA as a capture probe, Cao et al. demonstrated the proof of concept of a fluoroacetamide aptasensor by immobilizing the fluoroacetamide analog on a microplate to capture the aptamer sequences in place. The fluoroacetamide analyte competed with its immobilized analog to bind to and strip off the aptamers from the microwell into the supernatant, which could be taken to easily monitor fluorescence intensity [47].

### 5.3. Electrochemical Aptasensors

Electrochemical aptasensors are a kind of biosensor that measures the electrical signal changes as a result of aptamer-target interaction. They have gained much attention and are being developed for pesticide monitoring because of their high sensitivity, rapidity, simplicity, portability, miniaturizability and low cost. The current advancement of electrochemical pesticide aptasensors is mainly to improve the detection sensitivity by integrating aptamers with innovative nanocomposite materials and redox probes to enhance specific surface area and electron-transfer efficiency of the sensing electrode, among other techniques. A couple of key factors need to be figured out for developing electrochemical aptasensors, including aptamer immobilization, signal amplification and sensing strategies. This information is very important to decide on a transducer platform and its appropriate modification process. Besides the electrical circuit design, one major strategy to amplify the electrochemical signal is to increase the sensing area to load more aptamers and redox probes in order to recognize more target and generate more redox activity.

Several inorganic and biological materials have been successfully used to boost specific surface areas of the sensor interface. Chitosan is frequently exploited for modification of the electrode surface because of its three-dimensional cross-linking structure, which provide very large specific surface areas and has many amino and hydroxyl moieties essential for immobilization of aptamers and redox active materials [114]. Likewise, metal- and carbon-based nanomaterials offer great benefits for electrode modification; the most commonly found are AuNPs, AgNPs, MNPs, SWNCNTs, MWCNTs and GO. They improve not only surface areas but also electrical conductivity and may provide electrocatalytic activity of the sensor, where the electrode surface has been regularly modified by a combination of several ingredients, such as chitosan–iron oxide hybrid (Chit-IO) deposited on fluorine tin oxide electrode [115], graphene nanoplatelets/carboxylated multiwalled carbon nanotube nanocomposite doped with vanadium disulfide quantum dots (VS_2_QDs-GNP/MWCNTs) on glassy carbon electrodes [116] or reduced graphene oxide–silver nanoparticles and Prussian blue–gold nanocomposites (rGo-AgNPs/PC-AuNPs) deposited on glassy carbon electrodes [117]. In addition, molecular imprinted polymer (MIP) [40,118] and polydimethylsiloxane (PDMS) [119] film have been used to improve selectivity against pesticide targets and to introduce hydrophobicity for reusability of the sensing unit, respectively.

Most electrochemical pesticide aptasensors rely on a heterogeneous platform, which requires immobilizing aptamers on the sensing interface, especially via covalent bonding, affinity attachment, physical adsorption and self-assembling processes [120]. Covalent immobilization can be achieved by chemical coupling among functional groups labelled at the 5′ or 3′ end of aptamers and the corresponding chemical groups of an electrode surface, which are commonly amino, hydroxyl and carboxylic groups. Despite direct immobilization by covalent bonding, aptamers can be tethered on the surface via specific affinity molecules such as biotin-streptavidin and capture probe systems [121]. Moreover, thiolated aptamers can be easily functionalized onto gold-modified electrodes by the gold–sulfur self-assembling mechanism [122]. The electrochemical determination of aptamer–pesticide interaction, in either homogeneous or heterogeneous format, mostly relies on cyclic voltammetry (CV), differential pulse voltammetry and electrical impedance spectrometry (EIS); detail descriptions of these electrochemical approaches are beyond the scope of this review and can be explored elsewhere [123,124]. We mainly focus on the art of transforming the aptamer–pesticide complex to a measurable electrical signal, which is mainly dependent on how electrochemical redox probes get involved in the sensing system, as summarized below and in Table 4.

#### 5.3.1. Conjugation of Redox Probe to Aptamer or Molecular Probe

**Figure 6:**
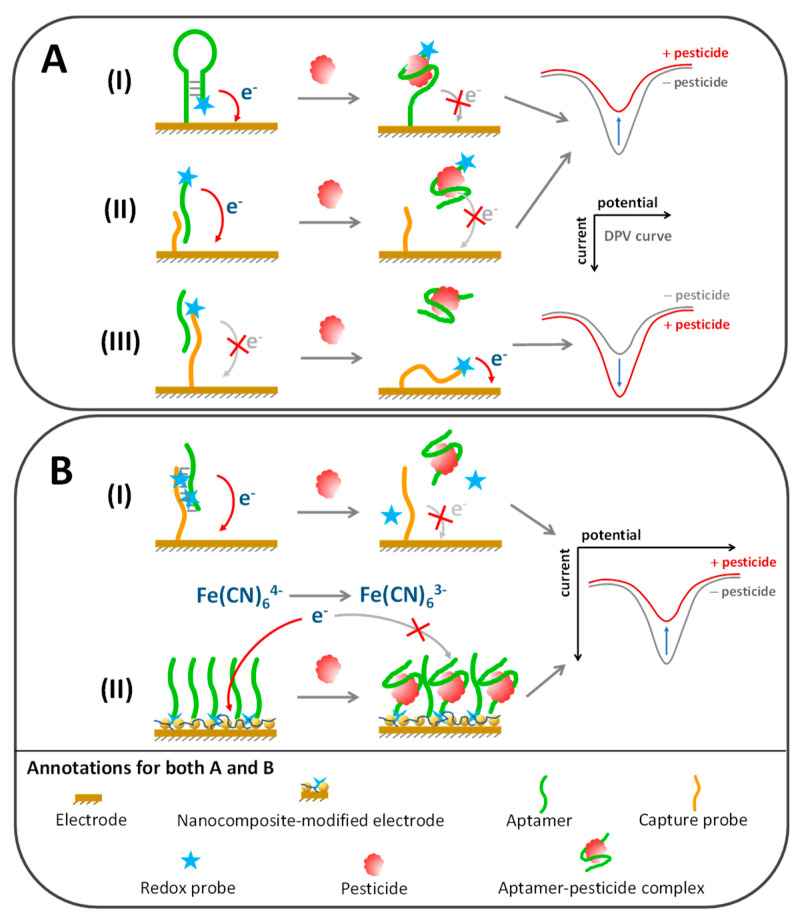
Schematic representation of redox probe-based pesticide detection. (**A**) Labeling strategies in electrochemical pesticide aptasensors: redox probe-labeled hairpin-structure aptamer (I), redox probe labelled-aptamer bystander on capture sequence (II) and aptamer bystander on redox probe-labeled capture sequence (III). (**B**) Noncovalent incorporation of redox probe in electrochemical pesticide aptasensors: intercalation (I) and in situ incorporation of probe with sensing interface (II). (Adapted by permission from Springer Nature Customer Service Centre GmbH: Springer Nature [140], copyright 2020.).

Among various redox probes, ferrocene and methylene blue are frequently used to label the aptamers or capture sequences in electrochemical pesticide aptasensors. Binding of the pesticide target causes conformational changes of the aptamer, which causes the labelled redox probe to separate or approach the electrode surface, which respectively results in reduction or enhancement of the electrical signal, as schematically represented in Figure 6A. Fu et al. developed voltametric aptasensors to determine organophosphates (OPs). Chitosan–graphene oxide nanocomposite was coated on the electrode surface to improve conductivity and participate in ferrocene-labelled aptamer immobilization. Initially, without OPs, the immobilized aptamers underwent hairpin-loop formation and brought the labelled ferrocene in close proximity to the electrode (Figure 6(AI)). The addition of OPs opened up the hairpin loop and hindered the redox signal away from the electrode, which measured profenofos, phorate, isocarbophos and omethoate at LOD of 0.01, 0.1, 0.01 and 0.1 nM, respectively [135].

Various enzymes can serve as redox indicators owing to their efficient oxidation-reduction catalytic activity, such as horseradish peroxidase (HRP), alkaline phosphatase (ALP) and glucose oxidase (GOD). An electrochemical aptasensor for profenofos was successfully developed by immobilizing the capture probe against profenofos-specific aptamers on an electrode modified with gold nanoparticles/polyaniline composite film (Figure 6(AII)). The profenofos analyte and immobilized capture probe were competitively bound with the biotinylated aptamers. In the absence of profenofos, aptamers were captured on an electrode via the immobilized capture probe, which could be recognized by alkaline phosphatase-bearing streptavidin to produce a measurable redox signal because of the 1-napthyl phosphate substrate degradation. The differential pulse voltammetry (DPV) signal of this system provided a linear response between 0.1 and 10 µM with an LOD of 0.27 µM of profenofos [121]. In addition, Vent polymerase and T7 exonuclease enzymes were applied for construction of a profenofos aptasensor. Ferrocene-labelled aptamer recognized profenofos and left its 3′ terminus free for binding with primer and further polymerization by Vent polymerase. The T7 exonuclease later digested the newly synthesized dsDNA and released the ferrocene probe to attach on an electrode surface. Both Vent polymerase and T7 exonuclease helped to amplify the sensing signal, which was able to detect profenofos with an LOD of 0.01 ng/mL [132].

#### 5.3.2. Noncovalent Incorporation of Aptamer Duplex with Redox Probe

It is well known that aptamers can form duplex structures with complementary capture probes or self-hybridization, which can be easily scrutinized using intercalating probes, in particular methylene blue, Hoechst 33,258 or osmium bipyridine-based complex (Figure 6(BI)). Electrochemical responses can be detected when redox probes intercalate between the base pairs or into the minor grooves of an aptamer duplex, where this phenomenon can be diminished after aptamers lose their complementary partner to form complexes with the cognate target. The duplex length can be customized to be longer to increase the load of redox probe intercalation and improve analytical sensitivity [140]. An example of this strategy is an electrochemical aptasensor toward chlorpyrifos developed by Xu and coworkers, who immobilized the hybridization probe to capture chlorpyrifos aptamer onto a copper oxide nanoflower–single walled carbon nanotube composite-coated electrode [128]. The aptamer–probe duplex could be monitored by DPV upon addition of methylene blue as a redox probe to bind with the guanine base [141]. Upon complexation with chlorpyrifos, the aptamer was dissociated from the hybridization probe, causing methylene blue release from the sensing interface and resulting in a decreased DVP peak corresponding with the chlorpyrifos concentration.

Besides intercalation, some redox probes directly interact with the nucleotide composition of aptamers. For example, a phosphate backbone could precipitate sodium molybdate by redox reaction and produce a measurable redox current, which was exploited for the development of a label-free acetamiprid aptasensor by Yi and colleagues [125]. They constructed a porous glassy electrode consisting of gold, chitosan and reduced graphene oxide nanocomposite (Au/3D–CS/rGO/GCE) and functionalized with acetamiprid aptamers, which were later hybridized with complementary probes that had a self-assembled elongation property to enhance the redox signal after interacting with sodium molybdate. The square wave voltammetry of this platform proved that it had good sensitivity toward acetamiprid in tea samples with an LOD of 71.2 fM.

#### 5.3.3. Integration of Redox Probes in Aqueous or Nanocomposite Phase

In some circumstances, conjugation or intercalation of redox probes with aptamers can interfere with the aptamer–target affinity and require sophisticated and time-consuming processes. Therefore, redox probes have been directly added in a sensing cocktail or imprinted in situ in a sensing interface to overcome such limitations (Figure 6(BII)). The ferrocyanide–ferricyanide ([Fe(CN)_6_]^3−/4−^) redox couple is often utilized as a signal indicator by merely adding it in an aqueous phase of electrochemical aptasensors, especially in EIS and DPV sensing platforms. In addition, different redox active materials have been successfully doped in the nanocomposite interface of electrodes, such as ferrocene, Prussian blue and nickel hexacyanoferrate, to serve as in situ redox probes to improve the sensitivity and simplicity of the aptasensor platform [117,122,129]. The idea of this strategy is that the aptamers immobilized on the electrode surface have to undergo conformational changes and form complexes with the cognate target, which is a relatively rigid structure and later block electron transfer among the redox probes and an electrode unit.

For example, an electrochemical aptasensor for profenofos was developed by immobilizing aptamers on a gold nanoshell/MWCNT-modified electrode. The DPV measurement under the [Fe(CN)_6_]^3−/4−^ redox probe environment gave a linear response to profenofos in a range of 0.1–10,000 ng/mL with an LOD of 0.052 ng/mL [133]. A similar strategy was applied by Shi et al. to develop an acetamiprid aptasensor; however, the redox signal associated with the aptamer–acetamiprid complex was dual amplified by means of AgNP-doped reduced GO and Prussian blue–gold nanocomposites. The current peak of CV scanning was reduced in an acetamiprid concentration dependent manner with a linear detection range from 1 pM to 1 µM and an LOD of 0.3 pM [117]. Electrochemical aptasensors that were ultrasensitive to four organophosphorus insecticides were demonstrated using an aptamer-modified micro-interdigitated electrode chip as a sensing probe without the need for nanocomposite modification. The interfacial capacitance over the aptamer-modified electrode was measured and it was found that its change rate (dC/dt) decreased linearly with an increased OP concentration, ranging around fM–nM. The LOD was found to be 0.38, 0.24, 1.67 and 0.34 fM for isocarbophos, profenofos, omethoate and phorate, respectively [139].

### 5.4. Electrochemiluminescence-Based Aptasensors

An electrochemiluminescence (ECL) aptasensor is a hybrid that combines luminescence and electrochemical phenomena, providing superb benefits for analytical applications. It translates an electrochemical event related to the aptamer–target interaction into a luminescence signal, which is quite sensitive because it glows in the dark and is free of photoexcitation interference, as found in fluorometry. Accomplishing an ECL aptasensor requires the redox-active luminescence probe to incorporate in an aptamer-modified electrode by either labelling to the aptamer sequence or affixing in the nanocomposite interface. For example, a carbofuran aptasensor was developed based on the electrochemiluminescence energy transfer (ECERT) event between fullerene-loaded gold nanoparticles (C60-AuNPs) and carbon dot-tagged aptamers, which were modified on an electrode surface to serve as electron donor and acceptor, respectively. Under a certain voltage and in the absence of carbofuran, the ECL signal of the carbon dot-labelled aptamer was enhanced by the C60-AuNP nanocomposite. The mechanism to explain this was that some electrons were donated from AuNPs through the superconducting property of C60 to activate carbon dot luminescence. On the contrary, the ECL emission was otherwise diminished with the addition of carbofuran because the compact structure of the aptamer–carbofuran complex caused a blockade of electron transfer between the electrode interface and carbon dot. A linear ECL response could be obtained in carbofuran in the range of 20 pM–2 nM with an LOD of 0.88 pM [41].

Likewise, an electrochemiluminescence-based aldicarb aptasensor was established by Li and colleagues. Instead of directly modified AuNPs on an electrode, AuNPs were rather tagged to the aptamer tail, while the electrode surface was modified with [Ru(bpy)_3_]^2+^-labelled dendritic poly(l-arginine) to capture the aldicarb-specific aptamer in place. Once they were bound with aldicarb, the AuNP-tagged aptamers underwent conformational rearrangement and disengaged from the arginine-rich polymer, resulting in ECL signal reduction, since the AuNPs were unable to influence the electronic energy transition of the [Ru(bpy)_3_]^2+^ modified on the electrode. This system could determine aldicarb down to an LOD of 9.6 pM [34]. In contrast to ECL assay, photoelectrochemical-based aptasensors utilize photo energy to activate the electron discharge of the light-responsive material in response to the aptamer–target complex. Recently, Ding et al. reported that visible light-responsive MoS_2_ nanosheets decorated with zinc phthalocyanine nanoparticles (ZnPc_2_/n-MoS_2_) could be applied to detect edifenphos by modification with the edifenphos-specific aptamer. In the absence of an edifenphos target, the immobilized aptamer sterically hindered electron transfer from the light-responsive element toward the electrode, where the current flow could be resumed in the presence of edifenphos. A linear detection range of edifenphos at 5 ng/L–10 µg/L with an LOD of 1.667 ng/L was demonstrated [142]. Detection characteristics of electrochemiluminescence aptasensors are summarized in Table 5.

### 5.5. Surface-Enhanced Raman Spectrometry-Based Aptasensors

Surface-enhanced Raman spectrometry (SERS) is a measurement of Raman scattering on a rough surface, like a metallic nanoparticle-coated surface, which is exquisitely sensitive to the plasmonic changes as a result of molecular adsorption on the surface. SERS is widely applied for biosensor development because it offers several advantages, such as high sensitivity, multiple-target detection capability and label-free analysis [144,150]. Even though SERS has been proven to be able to detect a single molecule adsorbed on a colloidal metal surface [151], functionalization of the SERS substrate with the target recognition element and Raman scattering enhancer remains important for its specificity and sensitivity to analytes.

An AuNP-modified SERS substrate bearing anti-malathion aptamers could detect malathion down to 3.3 µg/mL by means of a Raman microscope [49]. Another SERS-based malathion aptasensor was discovered by Nie and co-workers, who used silver colloidal coated with positively charged spermine to capture the malathion-specific aptamer on a substrate. In order to enhance the SERS signal, the colloidal substrate was induced to aggregate by the addition of Na^+^ salt. This system could simultaneously distinguish malathion from other pesticide interference based on SERS spectrum analysis, which quantitated malathion in a range of 0.5–10 µM [143]. In addition, SERS-based aptasensors provide an advantage in multiplex analysis of pesticides, as shown by Pang and colleagues [144]. An organophosphorus aptamer-functionalized silver dendritic nanoparticle substrate was developed for multiple detection of isocarbophos, omethoate, phorate and profenofos. Each kind of organophosphorus gave a distinct fingerprint of the SERS spectrum under a DXR Raman microscope with TQ Analyst software, which could discriminate and quantify them at the micromolar level using principal component analysis (PCA).

### 5.6. Miscellaneous Approaches

**Figure 7:**
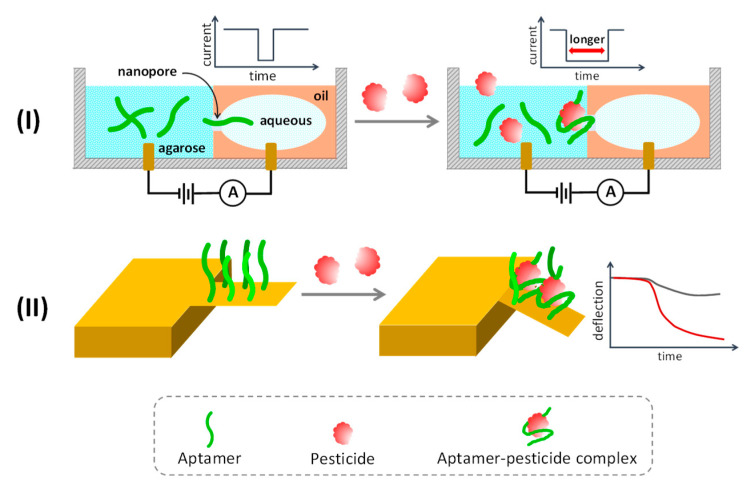
Ionic current-based aptasensors (**I**) (adapted with permission from Reference [147], copyright 2017 Royal Society of Chemistry) and microcantilever-based aptasensors (**II**) (adapted with permission from Reference [148] copyright 2018 Elsevier) for pesticide detection.

Apart from the above-mentioned techniques, over the last few years, some innovative approaches have been exploited for the development of pesticide aptasensors. A homogeneous assay of insecticide organophosphates was established by the use of broad-spectrum organophosphate-specific aptamers and capillary electrophoresis with a laser-induced fluorescence sensing unit (CE–LIF). Aptamers bound with their QD-labelled complementary sequence displayed distinct fluorescence peaks on CE chromatograms. Upon binding with organophosphorus targets, aptamers transformed their structure and released the complementary sequence to produce another specific fluorescent peak on the chromatogram. The fluorescence ratio of those two peaks implied the organophosphorus concentration, which was able to detect phorate, profenofos, isocarbophos and omethoate at an LOD of 0.20, 0.10, 0.17 and 0.23 μM, respectively [149]. Furthermore, omethoate vapor could be detected by the nanopore sensing system, which is label-free technology that can detect a single molecule by measuring the ionic current travelling through the nanoaperture between two isolated compartments (Figure 7I). Lipid bilayer holding the nanopore was used to separate the agarose chamber and aqueous-in-oil compartment. Free aptamers could translocate smoothly through the nanopore from cathodic agarose to anodic aqueous. Omethoate vapor diffused in an agarose compartment bound with the aptamers, interrupting the ionic current across the nanopores due to the blockage of nanopores by the rigid structure of the omethoate–aptamer duplex. This system could detect omethoate down to 4.8 nM in solution and 100 ppb as vapor with a short measurement time at about 60 s [147].

Aptamer-functionalized cellulose paper was used for selective extraction of acetamiprid and codeine and later served as a paper spray ionization source for ion mobility spectrometry. This system provided a seamless process of sample preparation and analysis that could selectively capture, desorb and ionize the analytes using a continuous solvent flow. A linear detection range of 5–300 ng/mL and an LOD of 3.7 ng/mL were obtained for acetamiprid [146]. A microcantilever array was utilized to detect profenofos by functionalization with a thiolated profenofos-specific aptamer (Figure 7II). Binding of profenofos with the immobilized aptamer introduced compressive surface stress on the microcantilever, causing significant deflection of the laser beam aimed at the microcantilever surface. The level of microcantilever deflection indicated a concentration of profenofos of 5–1000 ng/mL, with an LOD as low as 1.3 ng/mL (3.5 nM) (Table 5) [148].

## 6. Benchmarking with the ASSURED Criteria

To effectively control and decrease the risk of pesticide contamination, simple, accurate and affordable methods are required to analyze pesticide residues in food-stuffs and environments on a routine basis and at field sites. The analytical tests should provide enough specificity with a detection limit below the MRL of the target pesticide and be operable in the area of investigation by personnel with minimal training at a reasonable cost and with limited resources. Currently, in the healthcare sector, the ASSURED concept is widely used as a benchmark for assessing an ideal point-of-care test (POCT). It was coined by the World Health Organization Sexually Transmitted Diseases Diagnostics Initiative as an acronym for affordable, sensitive, specific, user-friendly, rapid and robust, equipment-free and deliverable to end users [152,153]. The required characteristics of pesticide screening tests for the general public precisely comply with the ASSURED criteria. Thus, the achievement of current pesticide aptasensors in terms of meeting the ASSURED criteria has been appraised in this review, as shown in Table 6 and as follows:

Affordable: Frequent screening of pesticides in commodities and living areas is one of the key factors to limit their usage and reduce the risk. So, the cost of testing and sample processing should be reasonable for routine operation by authorities, farmers and end consumers, especially in developing countries, where large quantities of pesticides are applied. The standard mass spectrometry-based method for pesticide quantification has very high cost and is generally accessible only by regulatory authorities or corporations. However, there are limited studies suggesting the appropriate cost of pesticide screening depending on socioeconomic status in different areas. Based on the authors’ opinion, the test should cost less than USD 2.00. Colorimetric-based aptasensors seem to be more affordable than others due to their simplicity of fabrication and resource requirements. Compared with other biosensors in the market, electrochemical aptasensors seem to have the potential to be affordable and usable if provided with a low-cost and pocket-size potentiostat.

Sensitive: The analytical methods must be sensitive enough to trace target pesticides at least at the tolerance limit level. The MRL depends on the type of pesticide and sample and varies among countries (readers are referred to the webpage of the regulatory agency of their country, such as the United States Environmental Protection Agency or the European Commission). For example, the European Union and Japan have adopted a default MRL of 0.01 mg/kg for pesticides that do not have a specific legal limit level, while it is 0.1 mg/kg for Canada and New Zealand [154], both of which can be uncovered by most of the present pesticide aptasensors. As shown in Table 2, Table 3, Table 4 and Table 5, electrochemical-based aptasensors mostly provide better sensitivity than other aptasensor platforms, with extremely low detection limits at the fM to pM level (equivalent to ppq to ppt, respectively). Optical aptasensors in both colorimetric and fluorometric approaches deliver comparable LODs at about the pM to nM range, while SERS-based aptasensors are unable to distinguish organophosphates (malathion, phorate, profenofos, isocarbofos and omethoate) at a default MRL of 10 ppb.

Specific: To avoid false positive results, aptasensors must accurately respond to the pesticide target without cross-reacting with other pesticides or interference. Almost all of the current pesticide aptasensors have been validated for excellent specificity toward their target and tolerance to interference in the sample matrix, so they seem to be eligible to serve as screening methods for pesticide residues in commodity and environmental samples. However, it needs to be kept in mind that each sensing modality also faces some challenges that might produce unwanted faulty results. For example, aptasensors based on colorimetric nanoparticle aggregation may be affected by the charged species present in the sample. Fluorescent probes are often sensitive to photobleaching and nonspecific quenchers that can influence the accuracy and stability of fluorescent-based aptasensors. Electrochemistry-based aptasensors can be prone to intervention by dissolved oxygen and temperature changes.

User-friendly: For successful adoption by farmers and the general public, the pesticide analysis should be simple to perform in a few steps with minimal training and should be easy to interpret. Nanoparticle-based colorimetric aptasensors usually require multiple steps of operation. The user needs to equilibrate the aptamers and nanoparticles with the sample, which later requires additional steps to induce nanoparticle aggregation for production of readout colors. The fluorometric approach is quite similar to the colorimetric process but mostly does not demand the additional steps to trigger signals. The electrochemical approach seems friendlier to end users because aptamers and signaling probes are often functionalized on the sensor chip, which can give readout signals by dropping the sample onto the sensors. Although it looks simple, current electrochemical pesticide aptasensors demand sophisticated instruments and experienced users for analysis because they are still in their infancy and need to be developed for the portable format. Other techniques, such as SERS, microcantilever and capillary electrophoresis, are quite difficult to operate by the general public.

Rapid and robust: The robustness and heat resistibility of aptamers have been widely appreciated and enable them to be handled under ambient conditions. An ideal POCT has been recommended to give results within 30 min and it should be able to operate and be stored at room temperature [155]. These characteristics can be applied to criticize rapid tests for pesticide analysis. Current pesticide aptasensors often take several minutes (20–60 min) to let the pesticide equilibrate with the aptamers and some aptasensors may require an hour to complete the whole process, especially in the colorimetric approach. Thus, the rapidity of pesticide aptasensors needs to be improved in the future, particularly by simplifying the procedure, transforming to a process without reagents and minimizing the sample contact time.

Equipment free: All current pesticide aptasensors greatly rely on the equipment to give interpretable results. Even if some colorimetric and fluorometric aptasensor readouts can be visualized by the naked eye, they still require at least a simple photometer or fluorometer for quantitative analysis. It is not the case that relying on equipment is not suitable for screening tests for pesticides but it depends on the level of application. If the aim is to use it in the laboratory to supplement the current standard chromatography-based methods, its superb accuracy may need to be compensated with a sophisticated device. However, if the intention is to use it in a field site or remote area, pesticide aptasensors should be portable, not require electricity and be battery operated. However, currently there is no significant evidence for the development of an apparatus toward strengthening the growth of aptasensors in pesticide analysis. Thus, this opportunity remains to be challenged. Commercially available low-cost photometers and fluorometers or smart phone cameras can simply be adopted to integrate with colorimetric aptasensors [107,156]. Many portable potentiostats are available on the market but they are quite expensive for the general public. Low-cost potentiostats with reasonable performance, like glucometers, could be devised to drive electrochemical aptasensors to market. Other aptasensor platforms, such as SERS, microcantilever and capillary electrophoresis, are in the early stages of development and currently inaccessible for general practice.

Deliverable to end users: To the best of our knowledge, none of the current pesticide aptasensors are commercialized or applied in real practice, so they would not be deliverable to end users. Besides, intensive validation of the prototypes against ISO-certified standard methods needs to be carried out before in-house use or delivery to end users.

Finally, even if nearly 100 innovative pesticide aptasensors have been established in various sensing platforms, most of them are in the proof-of-concept state and challenges remain for commercialization and application in real practice. The requirements of some sophisticated instruments and complex procedures are major barriers to currently developed pesticide aptasensors. Likewise, compliance with international standards (e.g., ISO and CE mark) also needs to be clarified. Among the current platforms of pesticide aptasensors, the colorimetric approach is closest to being applicable in real situations, because of its low cost, reasonable sensitivity and selectivity and easy interpretation by the naked eye and it can be done with a photometer or phone camera. For instance, one prototype operated on the lateral flow format and integrated with a smart phone camera to sense ppb level of chlorpyrifos, diazinon and malathion [107]. Meanwhile, electrochemical aptasensors are the most sensitive and simple to operate for pesticide screening but they still lack affordable equipment for end consumers, unless otherwise they can be incorporated with some low-cost and pocket-size potentiostats [157,158].

## 7. Conclusions and Outlook

Contamination of pesticides in the food chain and the living environments is a big challenge across the globe that needs to be routinely monitored to optimize pesticide usage, avoid health risk and maintain an ecological balance. Therefore, there is an urgent need for effective and user-friendly pesticide screening methods to cope with the limitations in routine operation of standard GC-MS and LC-MS methods, which require sophisticated and expensive equipment, complicated procedures, centralized laboratories and highly trained personnel. Aptamer-based biosensors or aptasensors, are a promising trend in pesticide screening because of their high sensitivity and specificity to pesticide targets along with robustness, cost-effectiveness and flexibility. However, in general, aptamers have been developed to recognize certain specific pesticides. There is a limited number of pesticide-specific aptamers and current aptamer-based methods have been developed to target only about 20 pesticides among several hundred variations. This inadequacy may hinder the ability of aptasensors to trace a wide range of pesticide residues that may be present in the sample.

Unlike chromatographic methods, aptasensors seem suitable for monitoring a few specific target pesticides at a time that we need to keep an eye on, such as aldicarb, carbendazim and paraquat, which have been banned or added to the watchlist in some countries [159]. Although a sequence for targeting four organophosphorus pesticides (phorate, profenofos, isocarbophos and omethoate) has been demonstrated [51], aptamers against multiple pesticides remain to be developed in the future in order to improve the multiplex analysis capability of aptasensors. Unfortunately, the traditional SELEX process for aptamer development is time-consuming and requires a lot of effort, so a high-throughput procedure for aptamer selection and characterization is important to accelerate the advancement of aptamer-based technology. Currently, high-throughput sequencing (HTS) together with high-performance bioinformatics computing help to reduce the number of selection rounds of SELEX and suggest candidate aptamers from a massive amount of enriched oligo sequences. Excellent reviews on the high-throughput process framework and bioinformatics tools for aptamer selection can be found elsewhere [160,161,162]. An automated platform for high-throughput aptamer screening has also been demonstrated by Wu and colleagues [163]. Nevertheless, to the best of our knowledge, there is still no systematic implementation of a high-throughput process in the development of aptamers against pesticides, so this remains to be explored in the near future to make progress.

Naturally, most pesticides are small molecules, making them difficult to sense and differentiate from other interference. Thanks to their excellent affinity and selectivity, aptamers have been successfully applied to sense various small molecules such as heavy metals, toxins, antibiotics, molecular makers and pesticides. Aptamers have been proven to be able to distinguish very similar molecules that differ only by enantiomeric or one functional group variation [164]. The current aptasensors as reviewed above can effectively detect trace amounts of pesticide residues with LODs ranging from the femtomolar to the micromolar level, depending on the aptamer sequence and its relevant pesticide as well as the sensing principle, in which most aptasensor candidates can achieve the common tolerance limits (MRLs) of pesticides. With continuing advances in computational power and in silico tools, the rational design of potential aptamers will make it possible to improve their affinity and selectivity toward cognate targets and save time and cost in aptamer production [165,166]. Indeed, it can be envisioned that the progress in aptamer development along with advances in biosensors and related technologies, such as nanomaterials, microelectronics and microfluidics, will enhance the sensitivity, specificity, multiplex ability, portability and cost-effectiveness of aptasensors to serve in routine screening and pesticide control in the near future. The authors believe that an ideal screening test for pesticides should be designed as a self-contained format without the need for additional reagents and it should be self-powered, light-weight and portable, with multiple target detection, affordable in terms of acquisition and maintenance and have a process that is simple to operate by personnel with minimal training. These characteristics are essential for the general public to adopt such tests to manage and control pesticides in remote areas, farms and households.

## Figures and Tables

**Table 1:** Available aptamer sequences for specific targeted pesticides.

Pesticide	Group	Selection Approach	Aptamer Sequence	*K_D_*	Ref.
Acetamiprid	Insecticide	Immobilization of library on beads via capture probe (Capture-SELEX)	TGTAATTTGTCTGCAGCGATTCTTGATCGCTGACACCATATTATGAAGA	4.98 µM	[33]
Aldicarb	Insecticide	N.A.	CCGGTGGGTGGTCAGCACCTGGGGGAGTCCGGATATGGCCCAGCGCATCACCAGTTCGCAAGC	N.A.	[34]
Atrazine	Herbicide	Immobilization of biotinylated-target on streptavidin-modified magnetic beads	TGTACCGTCTGAGCGATTCGTACGAACGGCTTTGTACTGTTTGCACTGGCGGATTTAGCCAGTCAGTGTTAAGGAGTGC	0.62 nM	[35]
		Capture-SELEX	TGTACCGTCTGAGCGATTCGTACTTTATTCGGGAAGGGTATCAGCGGGGTTCAACAAGCCAGTCAGTGTTAAGGAGTGC	N.A.	[36]
		Truncation of sequence obtained from capture-SELEX	ACCGTCTGAGCGATTCGTACTTTATTCGGGAAGGGTATCAGCGGGG	3.7 nM	
		Capillary electrophoresis-SELEX	CTACGCTAGCTTGTATGCCCATCTGACCTCTGTGCTGCTA	890 nM	[37]
Bromacil	Herbicide	Immobilization of biotinylated-target on streptavidin-modified magnetic beads	TGTACCGTCTGAGCGATTCGTACTGTGGGCACCAATCGTACCCAATACTTGCGAATCAGCCAGTCAGTGTTAAGGAGTGC	9.6 nM	[38]
Carbendazim	Fungicide	Immobilization of target-conjugated BSA on microplate	CGACACAGCGGAGGCCACCCGCCCACCAGCCCCTGCAGCTCCTGTACCTGTGTGTGTG	60.2 nM	[39]
			GGGCACACAACAACCGATGGTCCAGCCACCCGAATGACCAGCCCACCCGCCACCCCGCG	65 nM	
Carbofuran	Insecticide	N.A.	CACCTGGGGGAGTATTGCGGAGGAAAGAGAACACTGGGGCAGATATGGGCCAGCAGGTC	N.A.	[40,41]
Chlorpyrifos	Insecticide	Immobilization of biotinylated-target on streptavidin-resin beads	CCTGCCACGCTCCGCAAGCTTAGGGTTACGCCTGCAGCGATTCTTGATCGCGCTGCTGGTAATCCTTCTTTAAGCTTGGCACCCGCATCGT	N.A.	[42]
Diazinon	Insecticide	N.A.	ATCCGTCACACCTGCTCTAATATAGAGGTATTGCTCTTGGACAAGGTACAGGGATGGTGTTGGCTCCCGTAT	N.A.	[43]
		Computational screening of Bruno’s reported sequences [43]	ATCCGTCACACCTGCTCTAATATAGAGGTATTGCTCTTGGACAAGGTACAGGGATGGTGTTGGCTCCCGTAT	55.51 µM *	[44]
Dichlorvos	Insecticide	GOLD SELEX	GGAAGAACATGTGAGCAAAAGGCCAGCAAAAGGCCAGGAACCGTAAAAAGGCCGCGTTGCTGGCG	0.85 µM	[45]
Fipronil	Insecticide	Immobilization of target on magnetic beads via covalent coupling	TGTACCGTCTGAGCGATTCGTACAGTTTCTGGAGGACTGGGCGGGGTGACGGTTATAAGCCAGTCAGTGTTAAGGAGTGC	48 nM	[46]
Fluoroacetamide	Rodenticide	Immobilization of target-conjugated BSA on microplate	ACCTGCAGGCGCGAGTTTCAGATCAAAACTTGTCTGGCGT	1 µM	[47]
Imidacloprid	Insecticide	GO-SELEX	TGTCGTCTACGGTTTTGGTTGTTGTTTGTTGGTGGGTGTA	−2.86 kcal/mol ^†^	[48]
			GGTGTGTTTGTTGTTGTTCTTGGCTGGTTTTTCTTCCTG	−6.41 kcal/mol ^†^	
Malathion	Insecticide	Immobilization of target on PharmaLink affinity column	ATCCGTCACACCTGCTCTTATACACAATTGTTTTTCTCTTAACTTCTTGACTGCTGGTGTTGGCTCCCGTAT	N.A.	[49]
Dual targets: edifenphos (ED) and iprobenfos (IP)	Insecticide	GO-SELEX	CGTACGGAATTCGCTAGCTAAGGGATTCCTGTAGAAGGAGCAGTCTGGATCCGAGCTCCACGTG	ED = 38 nM	[50]
				IP = 1.67 µM	
Multiple targets (4OPs): phorate (PR), profenofos (PF), isocarbophos (IS) and omethoate (OM)	Insecticide	Capture-SELEX	AAGCTTTTTTGACTGACTGCAGCGATTCTTGATCGCCACGGTCTGGAAAAAGAG	PR = 1.43 µM	[51]
				PF = 1.25 µM	
				IS = 0.9 µM	
				OM = 2 µM	
			AAGCTTGCTTTATAGCCTGCAGCGATTCTTGATCGGAAAAGGCTGAGAGCTACGC	PR = 1.11 µM	
				PF = 1 µM	
				IS = 0.83 µM	
				OM = 2.5 µM	
		In silico design and truncation of above-mentioned sequence	AGCTTGCTGCAGCGATTCTTGATCGCCACAGAGCT	N.A.	[52]

*K_D_*, dissociation constant; N.A., not available; BSA, bovine serum albumin. * Inhibition constant (*K_i_*) value determined by computational analysis. ^†^ Aptamer sequence was suggested based on predicted Gibbs free energy (ΔG°).

**Table 2:** Colorimetric aptasensors for pesticide detection. (LOD, limit of detection).

Pesticide	Method	LOD	Linear Range	Sample	Ref.
Acetamiprid	Colorimetry	5 nM	75 nM–7.5 µM	Soil	[67]
Acetamiprid	Colorimetry	0.56 nM	8.7–920 nM	Waste water, soil and cucumber	[89]
Carbendazim	Colorimetry	2.2 nM	2.2–500 nM	Water	[83]
Chlorpyrifos	Colorimetry	11.3 ppm	35–210 ppm	River water	[90]
Isocarbophos	Colorimetry	7.1 µg/L	50–500 µg/L	Chinese cabbage, brassica rape and lettuce leaves	[91]
Isocarbophos	Colorimetry	0.015 µg/L	0.25–1.5 µg/L	Sewage, farm land water and pond water	[92]
Diazinon	Colorimetry	17.903 nM	0.141–0.65 nM	N.A.	[44]
Malathion	Colorimetry	0.06 pM	0.5-1000 pM	Lake water and apple	[86]
Malathion	Colorimetry	1.94 pM	0.01–0.75 nM	Lake water and apple	[82]
Malathion	Colorimetry	1:00 pM 3 pM (in serum)	5 pM–10 nM	Spiked human serum	[93]
Malathion	Colorimetry	0.5 pM	0.01 nM–0.75 nM	Lake water, tap water and apple	[85]
Omethoate	Colorimetry	0.1 µM	0.1 µM–10 µM	Soil	[94]
Phorate	Colorimetry	0.012 ng/mL	0–25 µg/mL	Human blood	[95]
Iprobenfos (IP) and edifenphos (ED)	Colorimetry	10 nM (IB) and 5 nM (ED)	10–100 nM (IP), 5–25 nM (ED)	Paddy and polished rice	[50]

**Table 3:** Fluorescence-based aptasensors for pesticide detection.

Pesticide	Method	LOD	Linear Range	Sample	Ref.
Acetamiprid	Fluorometry	3.2 nM	50 nM–1000 nM	Adulterated tea	[96]
Carbendazim	Fluorometry	2.33 nM	2.33–800 nM	Water	[97]
Diazinon	Fluorometry	0.13 nM	1.05–206 nM	River water, apple and cucumber	[63]
Edifenphos	Fluorometry	1.3 × 10^−4^ mg/L	0.5–6 µg/mL	Surface water and rice	[98]
Carbofuran	Fluorometry	3.8 nM	5–600 nM	Tap water, cucumber, cabbage, kiwifruit and apple	[99]
Fipronil	Fluorometry	105 nM	5 nM–500 nM	River water	[46]
Fipronil	Fluorometry	53.8 ppb	25–300 ppb	Liquid egg	[100]
Isocarbophos	Fluorometry	10 nM	10–500 nM	Cabbage	[101]
Isocarbophos	Fluorometry	0.11 µg/L	0.25–1.5 µg/L	Sewage, farm land water and pond water	[92]
Isocarbophos	Phosphorescence	0.57 µg/L	5–160 µg/L	Chinese cabbage, brassica rape and lettuce leaves	[91]
Isocarbophos	Fluorometry	0.11 µg/L	0.25–1.5 µg/L	Sewage, farm land water and pond water	[92]
Malathion	Fluorometry	4:00 p.m.	0.01 nM–1 µM	Tap water, lake water, soil and orange juice	[102]
Profenofos	Fluorometry	0.21 ng/mL	0.5–100 ng/mL	Tap water, cabbage and milk	[103]
Omethoate	Fluorometry	0.041 µM and	0.1–17 nM and	Cabbage and river water	[104]
		0.029 pM by unpolarized and polarized fluorometry	0.1 pM–1 µM by unpolarized and polarized fluorometry		
Isocarbophos (IS) and profenofos (PF)	Fluorometry	11.4 µM (IS) and 14 µM (PF)	50–500 µM	Water	[105]
Trichorfon (TC), glyphosate (GP) and malathion (ML)	Fluorometry	72.20 ng/L (TC), 88.80 ng/L (GP) and 195.37 ng/L (ML)	0.1 µg/L–10 mg/L	Lettuce and carrot	[106]
Chlorpyrifos (CP), diazinon (DA) and malathion (ML)	Fluorometric-lateral flow strip	0.73 ng/mL (CP), 6.7 ng/mL (DA) and 0.74 ng/mL (ML)	1–5 ng/mL (CP), 2–4 ng/mL (DA), and 1–3 ng/mL (ML)	Maize, long bean, cauliflower, eggplant, oyster mushroom, shiitake mush-room, apple, orange, tomato, blueberry, spinach, lettuce and cabbage	[107]
4OPs	Fluorometry	LOQ values are 19.2 nM (PR), 13.4 nM (PF), 17.2 nM (IS) and 23.4 nM (OM)	0.01–10 mg/kg	Cabbage	[52]
4OPs	Fluorometry	0.384 µM (PR), 0.134 µM (PF), 0.035 µM (IS) and 2.35 µM (OM)	0.268–26.8 µM (PF) and 0.346–34.6 µM (IS); no obvious relationship for PR and OM.	Dried tangerine peel	[108]

**Table 4:** Electrochemical aptasensors for pesticide detection.

Pesticide	Method	LOD	Linear Range	Sample	Ref.
Acetamiprid	Voltammetry	71.2 fM	0.1 pM–0.1 µM	Tea	[125]
Acetamiprid	Voltammetry	0.3 pM	1 pM–1 µM	Lettuce and rape	[117]
Acetamiprid	Voltammetry	0.077 pM	0.1 pM–10 nM	Lettuce, cabbage and spinach	[126]
Aldicarb	Voltammetry	0.1 pM	0.25–250 pM	Lake and river water	[122]
Carbofuran	Voltammetry	67 pM	0.2–50 nM	Chinese cabbage, chili, lettuce, tomato, apple, banana, tangerine and watermelon	[40]
Carbofuran	Voltammetry	0.033 ng/mL	0.1 ng/mL–100 µg/mL	Cabbage, lettuce, leek and pakchoi	[127]
Carbofuran	Voltammetry	70 pg/mL	0.1–150 ng/mL	Apple, celery and cabbage	[128]
Carbofuran	Voltammetry	0.35 fM	1 fM–0.4 pM	Apple and lettuce	[118]
Carbofuran	Voltammetry	0.33 ng/mL	1 ng/mL–100 µg/mL	Lettuce, leek and pakchoi	[129]
Diazinon	Voltammetry	0.0169 nM	0.1–1000 nM	Plasma of Wistar rat	[64]
Diazinon	Voltammetry	0.11 fM (DPV) and 2 fM (EIS)	0.5 fM–10 nM (DPV)	Human serum, river water, soil, apple and lettuce	[116]
	and EIS		0.1 fM–10 nM (EIS)		
Malathion	Voltammetry	0.001 ng/mL	0.001–10 ng/mL	Lettuce and soil	[115]
Malathion	Voltammetry	0.5 ng/mL	0.5–600 ng/mL	Cauliflower and cabbage	[130]
Malathion	Voltammetry	0.5 fM	0.1 fM–0.1 µM	Lettuce	[131]
Profenofos	Voltammetry	0.01 ng/mL	0 to 6.5 ng/mL	Rape	[132]
Profenofos	Voltammetry	0.27 µM	0.1–10 µM	Pear juice	[121]
Profenofos	Voltammetry	0.052 ng/mL	0.1 ng/mL–100 µg/mL	Spinach, lettuce and cabbage	[133]
4OPs	Voltammetry	0.003 nM (PF), 0.3 nM (PR), 0.03 nM (IS), and 0.3 nM (OM)	1–1000 nM (PR), 0.01–100 nM (PF), 0.1–1000 nM (IS), and 1–500 nM (OM)	Rape and spinach	[134]
4OPs	Voltammetry	0.1 nM (PR), 0.01 nM (PF), 0.01 nM (IS), and 0.1 nM (OM)	0.01–1,000 nM (PF), 0.1–800 nM (PR), 0.01–1,000 nM (IS), and 0.1–100 nM (OM)	Rape, cabbage, spinach and baby cabbage	[135]
4OPs	Refreshable voltammetry	N.A.	Qualitative detection above 1 µM	Baby cabbage	[119]
Acetamiprid	EIS	6:00 pM	40 pM–1 µM	N.A.	[136]
Acetamiprid	EIS	1:00 pM	10 pM–100 nM	Water	[137]
Aldicarb	EIS	10:00 pM	100 pM–1 µM	Water	[137]
Aldicarb	EIS	40 pM	0.6 nM–1 µM	N.A.	[136]
Carbendazim	EIS	8.2 pg/mL	10 pg/mL–10 ng/mL	Mango juice, soya milk, tomato and plum	[39]
Carbendazim	EIS	0.5 pg/mL	1–1000 pg/mL	Lettuce and orange juice	[138]
4OPs	Capacitance	0.24 fM (PR), 0.34 fM (PF), 0.38 fM (IS) and 1.67 fM (OM)	3.84 fM–3.84 nM (PR), 2.68 fM–2.68 nM (PF), 3.46 fM–3.46 nM (IS) and 4.69 fM–4.69 nM (OM)	Lettuce	[139]

**Table 5:** Miscellaneous approaches with aptasensors for detection of pesticides.

Pesticide	Method	LOD	Linear Range	Sample	Ref.
Aldicarb	ECL	9.6 pM	40 pM–4 nM	Turnip, cabbage, potato, banana, celery and irrigation water	[34]
Carbofuran	ECL	0.88 pM	20 pM–8 nM	Cowpea, cabbage, chili, tomato, lettuce, banana, celery, carrot, capsicum and apple	[41]
Edifenphos	ECL	1.667 ng/L	5 ng/L–10 µg/L	Rice	[142]
Malathion	SERS	3.3 µg/mL	3.3–33.3 µg/mL	N.A.	[49]
Malathion	SERS	N.A.	0.5–10 µM	Tap water	[143]
4OPs	SERS	0.4 µM (PR), 14 µM (PF), 3.4 µM (IS), and 24 µM (OM)	0–3.8 mM (PR): not available for other pesticides	Apple juice	[144]
Acetamiprid	Resonance Light Scattering (RLS)	1.2 nM	0–100 nM	Lake water	[145]
Acetamiprid	Sample extraction in ion mobility spectrometry	1.8 ng/mL	5–300 ng/ml	Wastewater, tomato and cucumber	[146]
Omethoate	Ionic current measurement	4.8 nM (in solution) and 100 ppb (in vapor)	N.A.	N.A.	[147]
Profenofos	Microcantilever	1.3 ng/mL (3.5 nM)	5–1000 ng/mL	Chinese chive	[148]
4OPs	Fluorometric-capillary electrophoresis (CE)	0.20 µM (PR), 0.10 µM (PF), 0.17 µM (IS) and 0.23 µM (OM)	0.6–10 µM (PR), 0.3–10 µM (PF), 0.5–10 µM (IS) and 0.7–10 µM (OM)	Apple	[149]

**Table 6:** Achievement of ASSURED criteria of current pesticide aptasensors represented by three-scale rating. More plus signs (+) means higher degree of alignment with the criteria.

ASSURED	Sensing Principle					
	Colorimetry	Fluorometry	Electrochemistry	ECL	SERS	Others
Affordable	+++	++	++	+	+	+
Sensitive	++	++	+++	++	+	+
Specific	+++	+++	+++	+++	+++	+++
User-friendly	++	++	++	++	+	+
Rapid and robust	++	++	+++	++	++	+
Equipment-free	+++	++	+	+	+	+
Deliverable to end user	+	+	+	+	+	+
